# Cellular Activity Modulation Mediated by Near Infrared-Irradiated Polydopamine Nanoparticles: In Vitro and Ex Vivo Investigation

**DOI:** 10.1021/acsnano.5c04181

**Published:** 2025-04-24

**Authors:** Alessio Carmignani, Takeru Yamazaki, Matteo Battaglini, Cong Quang Vu, Attilio Marino, Seika Takayanagi-Kiya, Taketoshi Kiya, Andrea Armirotti, Andrea Di Fonzo, Satoshi Arai, Gianni Ciofani

**Affiliations:** a Istituto Italiano di Tecnologia, Smart Bio-Interfaces, Viale Rinaldo Piaggio 34, Pontedera 56025, Italy; b Kanazawa University, WPI Nano Life Science Institute, Kakuma-machi, Kanazawa 920-1192, Japan; c Kanazawa University, Graduate School of Natural Science & Technology, Kakuma-machi, Kanazawa 920-1192, Japan; d Analytical Chemistry Facility, Istituto Italiano di Tecnologia, Via Morego 30, Genova 16163, Italy

**Keywords:** polydopamine nanoparticles, photothermal stimulation, cell activity modulation, acetylcholine release, *Drosophila melanogaster*

## Abstract

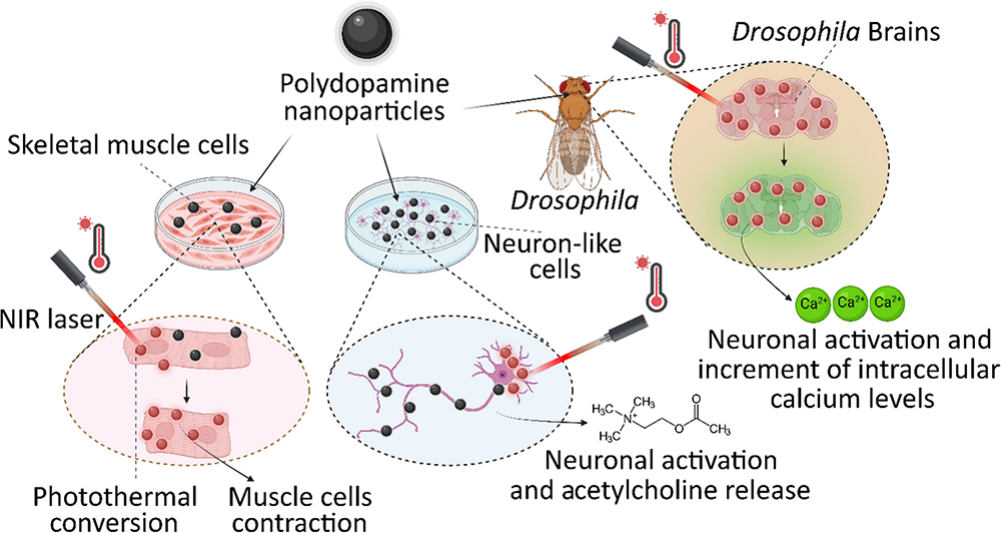

The precise control of cell activity is crucial for understanding and potentially treating many disorders. Focusing on neurons and myotubes, recent advancements in nanotechnology have introduced photoresponsive nanoparticles as an alternative tool for modulating cell function with high spatial and temporal resolution. This approach offers a noninvasive alternative to traditional stimulation techniques, reducing potential tissue damage and improving the specificity of cell activation. Here, we introduce an approach envisioning fully organic polydopamine nanoparticles (PDNPs) to remotely modulate the activity of differentiated SH-SY5Y cells and differentiated C2C12 cells, via near-infrared (NIR) laser stimulation. Confocal microscopy imaging revealed the possibility of thermally activating individual neuron-like cells, eliciting a significant cellular response characterized by the generation of calcium transients and the subsequent release of the neurotransmitter acetylcholine. Similarly, we demonstrated the possibility of precisely triggering the muscle contraction of single myotubes. Additionally, we investigated the antioxidant properties of PDNPs, demonstrating their capacity to prevent an increase in oxidative stress levels related to an increase in intracellular temperature. Moreover, proteomic analysis revealed that a PDNP treatment could positively affect neuronal plasticity and nervous system maturation, besides promoting muscle growth and preserving its functional integrity, underscoring its potential to support both neural and musculoskeletal development. Eventually, the effect of the NIR laser irradiation in the presence of PDNPs in neuron-like cells was successfully evaluated ex vivo on brains of *Drosophila melanogaster*, genetically modified to express the fluorescent calcium indicator jGCaMP7c.

The ability to remotely stimulate and control neuronal and muscle activity has always attracted considerable interest among researchers, from “traditional” biomedical applications such as the treatment of neurological disorders like Parkinson’s disease and spinal cord injuries,^1,2^ until exploitation in bionics and skeletal muscle tissue engineering.^3,4^ Several methods have been proposed so far to induce neuronal or muscular cell activation, including stimulation with ultrasound, magnetic fields, electric cues, or light.^5^

Therapeutic neuromodulation approaches can be categorized into invasive and noninvasive techniques. Invasive methods, such as intracranial cortical stimulation (ICS) and deep brain stimulation (DBS), offer high spatiotemporal precision but require surgical procedures for electrode implantation, often leading to subsequent on-site inflammation and gliosis.^6^ Noninvasive brain modulation techniques, like transcranial direct current stimulation (tDCS) and transcranial magnetic stimulation (TMS), enable the modulation of neuronal activity by applying external stimuli, such as electric current and magnetic fields, without the need for surgery.^7^ The clinical translation of these techniques is however strongly limited by their low spatial resolution.^8^

Regarding the activation of muscle cells, electric stimulation is currently the most widely used approach, not only to induce myotube contraction but also to promote and enhance myogenic differentiation.^9,10^ However, this technology has significant drawbacks that greatly limit its use, such as the lack of uniformity in the electric field generated by the electrodes and the adverse effects related to the release of toxic species due to electrolysis.^11^ This all considered, there is an urgent need for a more efficient, noninvasive, and targeted neuronal and muscular stimulation.

A noninvasive alternative could be represented by optogenetics, a powerful technique that enables precise control of cell activity through the expression of light-sensitive ion channels, such as channelrhodopsins.^12^ By using specific wavelengths of light, optogenetics allows for targeted stimulation of neurons and muscle fibers with high spatial and temporal resolution.^13^ However, its application is limited by the need for genetic modification, which requires viral vectors or transgenic models;^14^ additionally, optogenetic activation relies on visible light, which has limited tissue penetration, making in vivo applications challenging.^15^

The advancement of nanotechnology has significantly transformed the biomedical sector in various ways, and a deep impact has been provided by “smart” nanomaterials that, acting as nanotransducers, are able to convert one form of energy into another.^16^ Significant examples are provided by magnetic nanoparticles, such as iron oxide nanoparticles, which can be controlled using external magnetic fields to generate localized heating or induce electric currents,^17−20^ or, among others, piezoelectric nanoparticles, which generate electric charges in response to mechanical stress.^21−23^ However, magnetic and piezoelectric nanotransducers face several challenges related, in particular, to the intensity of magnetic fields or of mechanical stimulation that have been respectively applied. A suitable alternative is provided by light-responsive nanoparticles, mainly represented by gold-based,^24^ carbon-based,^25^ and polymeric^26^ systems, which have been explored for photothermal and photodynamic applications. Light-responsive nanotransucers are also investigated for cell activity modulation,^27,28^ mainly exploiting ultraviolet (UV),^29^ short-wavelength visible (Vis),^11,30^ and infrared (IR) light sources.^31^ However, the light at these wavelengths has limited tissue penetration; conversely, near-infrared (NIR) radiation can deeply enter into tissues, thanks to their relative transparency in this spectral window.^32^

The most common examples of nanotransducers capable of responding to NIR light for neuron and muscle stimulation are represented by gold-based nanomaterials.^33−35^ Thanks to their photothermal conversion properties, it is possible to precisely raise the intracellular temperature during NIR stimulation with the potential to reach thresholds for neuron or muscle activation. These findings have paved the way for several potential biomedical uses, however with the concerns of an overproduction of oxidative stress following the increment of temperature.^36^ Furthermore, the clinical use of metal-based nanoparticles is constrained because of their low biodegradability and prolonged material retention in tissues, particularly in the liver.^37,38^

Among the potential candidates as nanotransducers for NIR photothermal conversion, we selected polydopamine nanoparticles (PDNPs), widely exploited for their high biocompatibility, biodegradability, antioxidant properties, and effectiveness in photothermal conversion.^39−41^ Polydopamine owns abundant aromatic rings, allowing for the loading of drugs through π–π stacking and hydrogen-bonding interactions;^42^ additionally, polydopamine contains catechol and quinone reactive groups, facilitating its covalent attachment to compounds containing amino or sulfhydryl groups via Michael addition or Schiff base reactions.^43^

In this work, we aimed at thermally stimulating neuron-like cells and myotubes to promote the precise release of acetylcholine and the myosin activation in a noninvasive manner, while simultaneously preventing the increase in oxidative stress levels caused by the rise in intracellular temperature. Moreover, we also validated our approach in ex vivo experiments performed on brains from *Drosophila melanogaster*.

## Results

### Nanoparticle Characterization and Interactions with Cells

**Figure 1:**
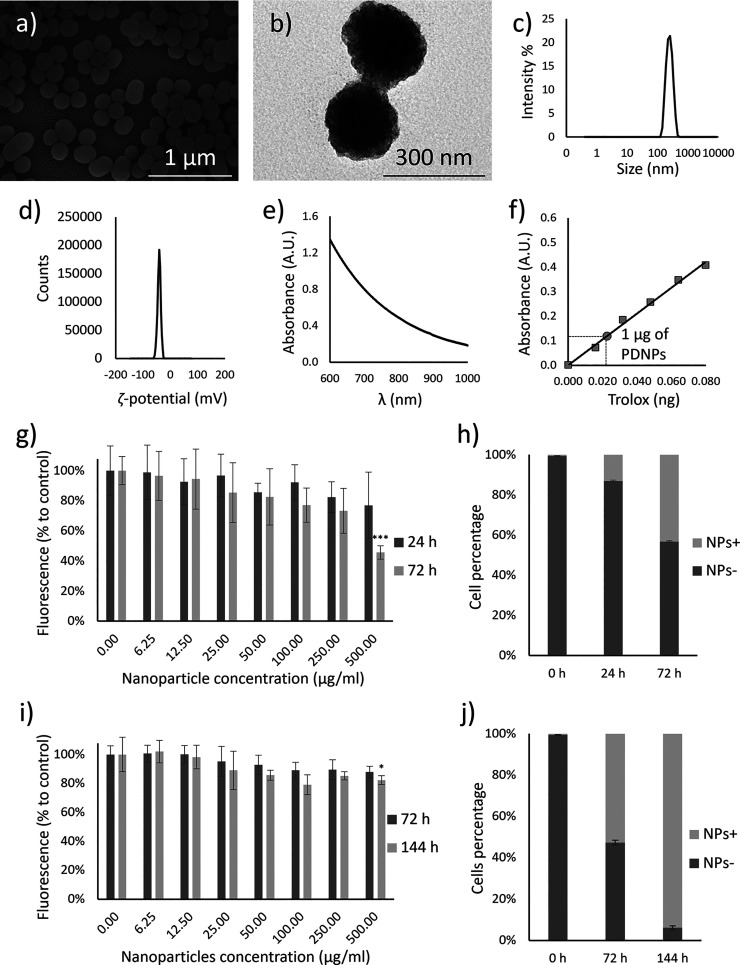
PDNP characterization and their interaction with SH-SY5Y and C2C12 cells. Representative (a) SEM and (b) TEM images. (c) Hydrodynamic diameter distribution and (d) ζ-potential analysis. (e) NIR absorption spectrum. (f) Trolox antioxidant activity standard curve, with highlighted a Trolox-equivalent antioxidant capacity of 1 μg of PDNP (gray dot). (g) PicoGreen assay on differentiated SH-SY5Y cells (*n* = 6, ***p < 0.001). (h) Flow cytometry analysis of PDNP internalization by differentiated SH-SY5Y cells. (i) PicoGreen assay on differentiated C2C12 cells (*n* = 6, *p < 0.05). (j) flow cytometry analysis of PDNP internalization by differentiated C2C12 cells.

Scanning electron microscopy (SEM, Figure 1a) and transmission electron microscopy (TEM, Figure 1b) images revealed spherical nanostructures with an average diameter of 197.5 ± 8.4 nm. Dynamic light scattering (DLS) assessments highlight a uniform size distribution of PDNPs, with hydrodynamic diameter (*D*_h_) and polydispersity index (PDI) values of 236.9 ± 11.1 and 0.03 ± 0.15, respectively (Figure 1c), along with a zeta potential (ζ-potential) of −40.4 ± 0.7 mV (Figure 1d). The stability assay results, obtained by dispersing PDNPs in cell culture medium, demonstrated that the nanoparticles remained stable throughout the entire observation period with no significant variations in *D*_h_ and PDI (Figure S1). The incubation of PDNPs in a solution of the lysosomal enzyme cathepsin B showed no significant short-term effects; however, after 6 days of incubation, a reduction of approximately 37% in *D*_h_ and a 24% increase in PDI were observed (Figure S2).

The NIR absorption spectrum (Figure 1e) shows a typical broad absorbance of PDNPs at these wavelengths. The antioxidant activity of PDNPs was assessed by determining the Trolox equivalents, an antioxidant compound commonly used as a reference, indicating that 1 μg of PDNPs is equivalent to 0.022 ng ± 0.003 of Trolox (Figure 1f).

Interactions between cells and nanoparticles were initially assessed by examining their impact on the cellular viability. SH-SY5Y cells were exposed to increasing concentrations of PDNPs (ranging from 0.00 to 500.00 μg/mL) and then evaluated after 24 and 72 h using the PicoGreen assay (Figure 1g). The measured fluorescence levels indicate the DNA content in the culture, which directly correlates with the cell count, thus enabling the assessment of the potential impact of the nanoparticles on the cell viability. After 24 h of treatment, none of the experimental conditions showed a statistically significant reduction in cell viability (*p* > 0.05). However, significant effects of PDNPs were observed after 72 h of treatment at the highest tested concentration (500.00 μg/mL; *p* < 0.001).

The impact of prolonged NIR laser stimulation on cell viability was also assessed under the same experimental conditions (Figure S3a). No statistically significant effects were observed after 24 h (*p* > 0.05); however, after 72 h of PDNP treatment and following laser stimulation, a significant reduction in viability was detected only at the highest tested concentration (500.00 μg/mL; *p* < 0.05).

Cellular uptake of DiO-PDNPs (100 μg/mL) by SH-SY5Y was assessed using flow cytometry (Figure 1h andFigure S3b) and confocal microscopy (Figure S3c). Both methods revealed a time-dependent internalization trend. Specifically, flow cytometry analysis showed that 13.1 ± 0.5% of cells were PDNP-positive after 24 h of incubation, increasing to 43.3 ± 0.4% after 72 h.

C2C12 cells were exposed to the same concentrations of PDNPs (ranging from 0.00 to 500.00 μg/mL) and then evaluated after 72 and 144 h using the PicoGreen assay (Figure 1i). After 72 h of treatment, none of the experimental conditions showed a statistically significant reduction in cell viability (*p* > 0.05), while after 144 h significant effects of PDNPs were observed at the highest concentration tested (500.00 μg/mL; *p* < 0.05). The effect of NIR laser stimulation was also evaluated in C2C12 cells (Figure S4a). After 72 h of exposure to PDNPs, no significant impact of laser stimulation on cell viability was observed (*p* > 0.05). However, after 144 h of treatment with PDNPs, a statistically significant reduction in viability was detected at the highest tested concentration (500.00 μg/mL; *p* < 0.01).

Cellular uptake by these cells, once again assessed through flow cytometry (Figures 1j and Figure S4b) and confocal microscopy (Figure S4c), displayed again a time-dependent internalization trend; in more detail, flow cytometry analysis showed that 52.6 ± 1.1% of cells were PDNP-positive after 72 h of incubation, increasing to 93.8 ± 0.9% after 144 h.

The colocalization of DiO-PDNPs with lysosomes and mitochondria was quantitatively analyzed using Pearson’s correlation coefficient. Representative confocal images illustrating the intracellular localization of DiO-PDNPs in differentiated SH-SY5Y cells are provided in Figure S5a. High levels of colocalization were observed in lysosomes (0.23 ± 0.04 after 24 h and 0.42 ± 0.14 after 72 h, Figure S5b); conversely, negligible colocalization was observed with mitochondria (0.04 ± 0.01 after 24 h and 0.19 ± 0.03 after 72 h). In Figure S6a, representative confocal images of the intracellular localization of DiO-PDNPs in differentiated C2C12 cells are reported. As in the previous case, also in myotubes, high levels of colocalization in lysosomes were observed (0.55 ± 0.01 after 72 h and 0.48 ± 0.04 after 144 h, Figure S6b); conversely, lower colocalization with mitochondria was highlighted (0.16 ± 0.03 after 72 h and 0.16 ± 0.04 after 144 h).

### PDNP Effects on Cell Differentiation

The ability of PDNPs to promote neurite outgrowth and differentiation in SH-SY5Y cells was evaluated by using epifluorescence microscopy (Figure S7a). Following 3 days of incubation with differentiation medium, SH-SY5Y cells exhibited a median neurite length of 19.5 ± 0.9 μm. Conversely, cells treated for 72 h with the same medium supplemented with 100 μg/mL of PDNPs displayed a statistically significant increase in neurite length, with a median of 39.0 ± 1.4 μm (*p* < 0.001, Figure S7b). In the absence of PDNPs, each differentiated SH-SY5Y cell showed a median number of neurites equal to 2.00 ± 0.88, whereas in the presence of nanoparticles, the median doubled to 4.00 ± 1.26 (*p* < 0.001, Figure S7c).

Myotube development in C2C12 cells promoted by PDNPs was evaluated with confocal microscopy (Figure S8a). At the end of 6 days of differentiation, untreated myotubes exhibited a median length of 205.3 ± 36.4 μm and a median width of 22.6 ± 7.0 μm, while PDNP-treated myotubes showed a median length of 195.5 ± 29.4 μm and a median width of 24.8 ± 5.6 μm (Figures S8b and S9c). In the absence of PDNPs, each myotube showed a median number of nuclei equal to 8.5 ± 2.9, whereas in the presence of nanoparticles, the median resulted 11.0 ± 3.2 (Figure S8d).

### Photothermal Stimulation of Individually Irradiated Cells

**Figure 2:**
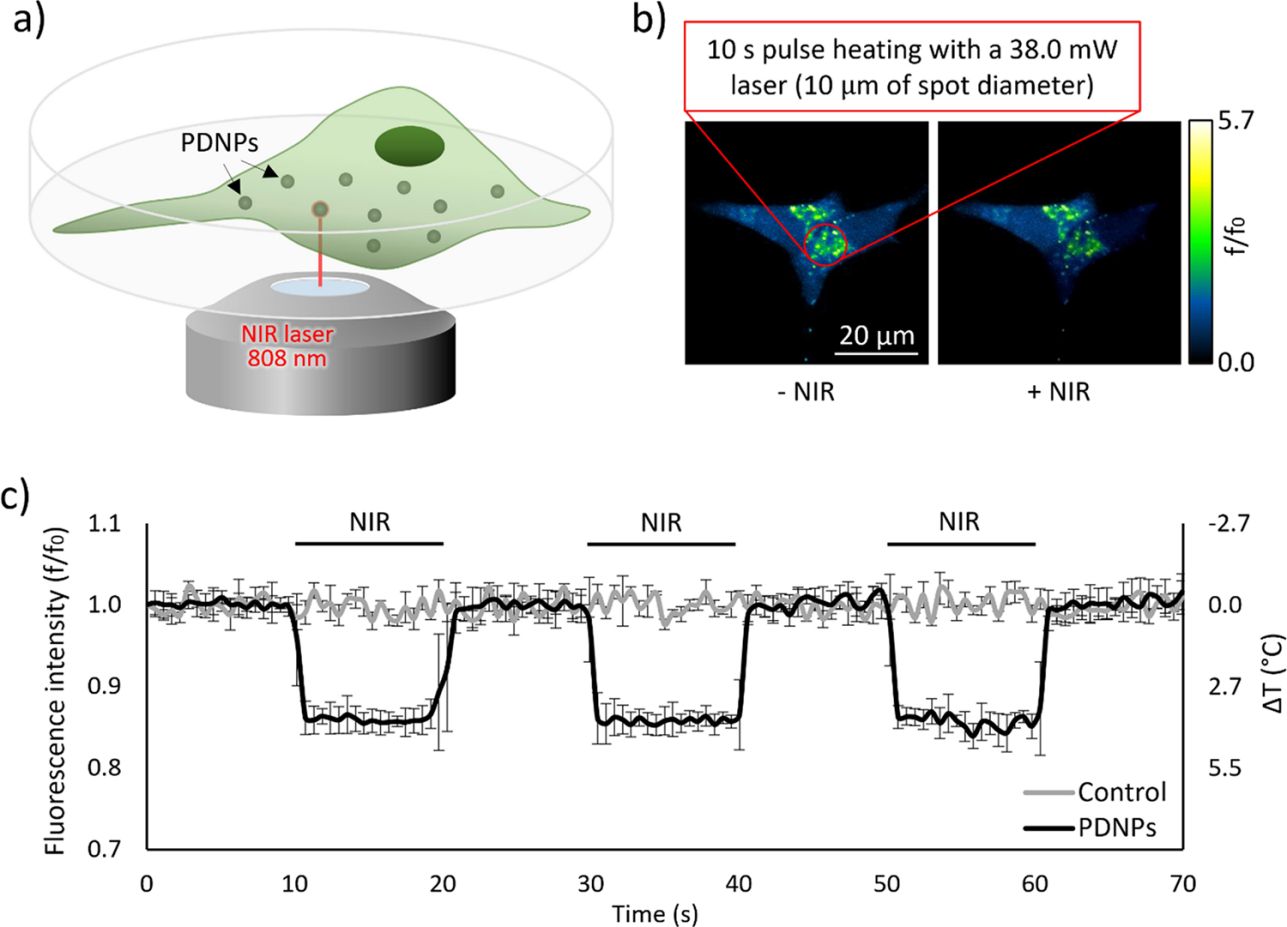
Neuronal stimulation analysis. (a) Schematization of the irradiation setup. (b) Representative acquisitions highlighting the thermosensitive dye fluorescence decrement during NIR laser irradiation of differentiated SH-SY5Y cells treated with PDNPs. (c) Temperature increment during the NIR stimulation.

Differentiated SH-SY5Y cells underwent stimulation using an NIR laser over a circular region with a diameter of 10 μm, employing 10 s of pulse irradiation with a power of 38.0 mW (setting schematization in Figure 2a). The increment in intracellular temperature was measured using confocal microscopy imaging and by exploiting a temperature-sensitive fluorescent dye belonging to the Thermo Greens family, compounds derived from the structure of fluorescent dyes known as BODIPY (Figure 2b).^44^ As depicted in Figure 2c, laser irradiation without PDNPs did not induce a significant decrement in cell fluorescence levels, indicating no increment in the temperature. Conversely, NIR irradiation in the presence of nanoparticles led to a reversible reduction in fluorescence levels, indicating a maximum intracellular temperature increment of approximately 4.4 °C, starting from 37 °C.

**Figure 3:**
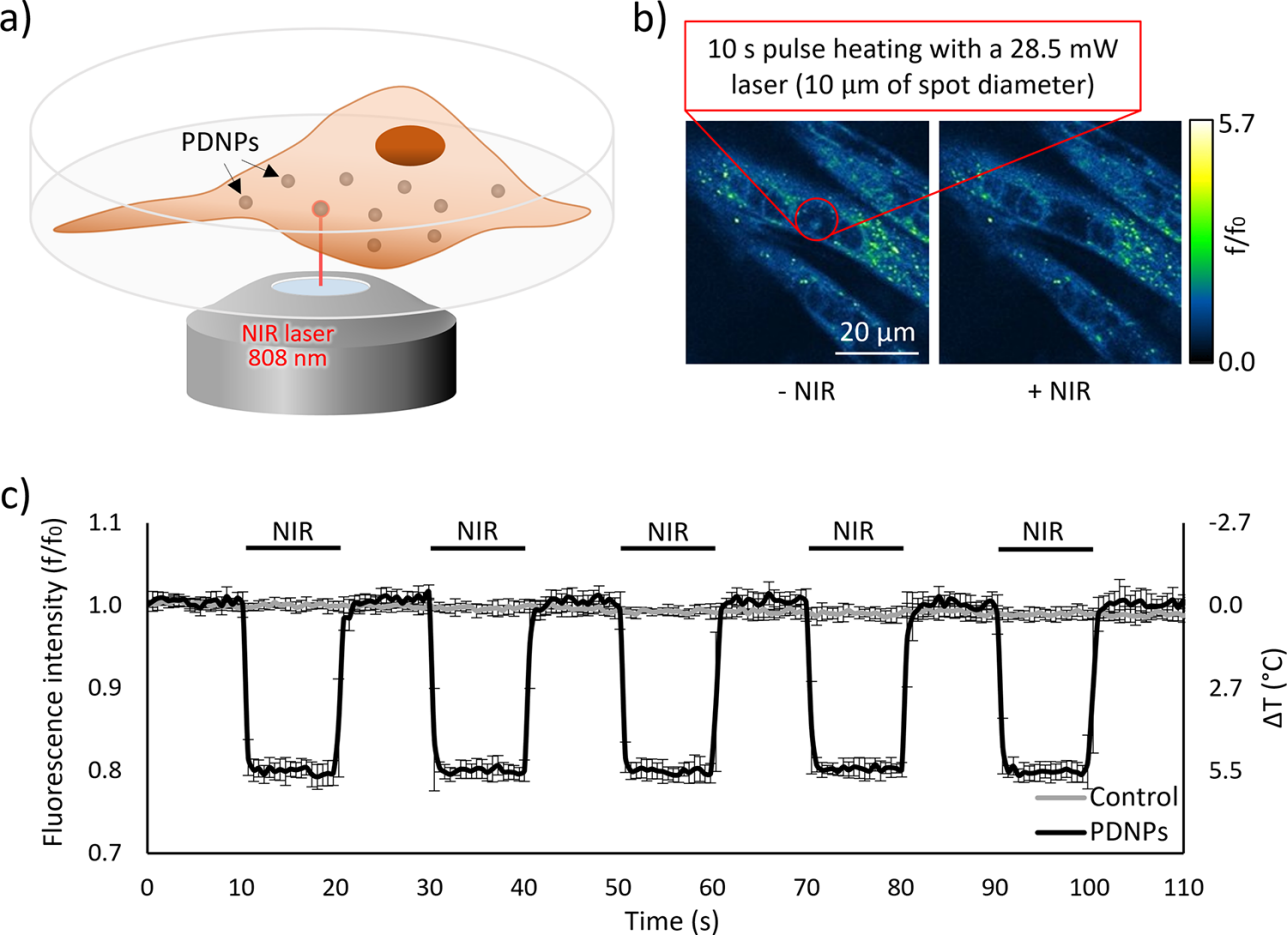
Myotubes stimulation analysis. (a) Schematization of the irradiation setup. (b) Representative acquisitions highlighting thermosensitive dye fluorescence decrement during NIR laser irradiation of differentiated C2C12 cells treated with PDNPs. (c) Temperature increment during the NIR stimulation.

Differentiated C2C12 cells underwent an analogous stimulation protocol (Figure 3a), and the increment in the intracellular temperature was again assessed by exploiting a temperature-sensitive dye (Figure 3b). In this case, a lower laser power (28.5 mW) was implemented, since a more pronounced temperature increase was detected, most probably because a higher PDNP internalization extends by C2C12 cells with respect to SH-SY5Y. As depicted in Figure 3c, laser irradiation without PDNPs did not induce a significant decrement in cell fluorescence levels even in myotubes, indicating no increment in temperature. On the other hand, NIR laser irradiation of PDNP-treated cells led to a maximum intracellular temperature increment of approximately 5.7 °C, starting from 37 °C.

### Neuronal Activation Assessment

The ability of PDNPs to induce the activation of single neuron-like cells upon NIR laser irradiation was investigated through calcium imaging. Differentiated SH-SY5Y cells were exposed to laser irradiation (50 ms; Figure S9a), and as depicted in Figure S9b, stimulation in the absence of PDNPs did not result in any significant change in fluorescence levels (due to the calcium indicator Fluo-4 AM), indicating no increase of intracellular Ca^2+^ levels. Conversely, stimulation of PDNP-treated SH-SY5Y cells with NIR laser-induced the production of a calcium transient, leading to a maximum fluorescence increment of 32.8 ± 9.6%.

The role of calcium channels in the effect produced by the stimulation was investigated by using lanthanum chloride (LaCl_3_), a widely used calcium channel blocker.^45^ In the presence of 50 μM of LaCl_3_ no fluorescence increment was observed (Figure S9b), suggesting the involvement of calcium channels in the observed Ca^2+^ level elevation. The sources of Ca^2+^ involved in the stimulations, such as intracellular stores and/or the extracellular environment, were investigated as well (Figure S9b). NIR stimulation of PDNP-treated cells conducted in Ca^2+^-free conditions and in the presence of 5 mM of the Ca^2+^-chelator ethylene glycol tetraacetic acid (EGTA) produced a maximum fluorescence increment of 10.5 ± 3.5%, while laser stimulation following the depletion of Ca^2+^ stored within the endoplasmic reticulum (ER) using thapsigargin, and, in the presence of PDNPs, led to the generation of a calcium transient indicated by a 22.9 ± 3.1% increment in fluorescence levels.

**Figure 4:**
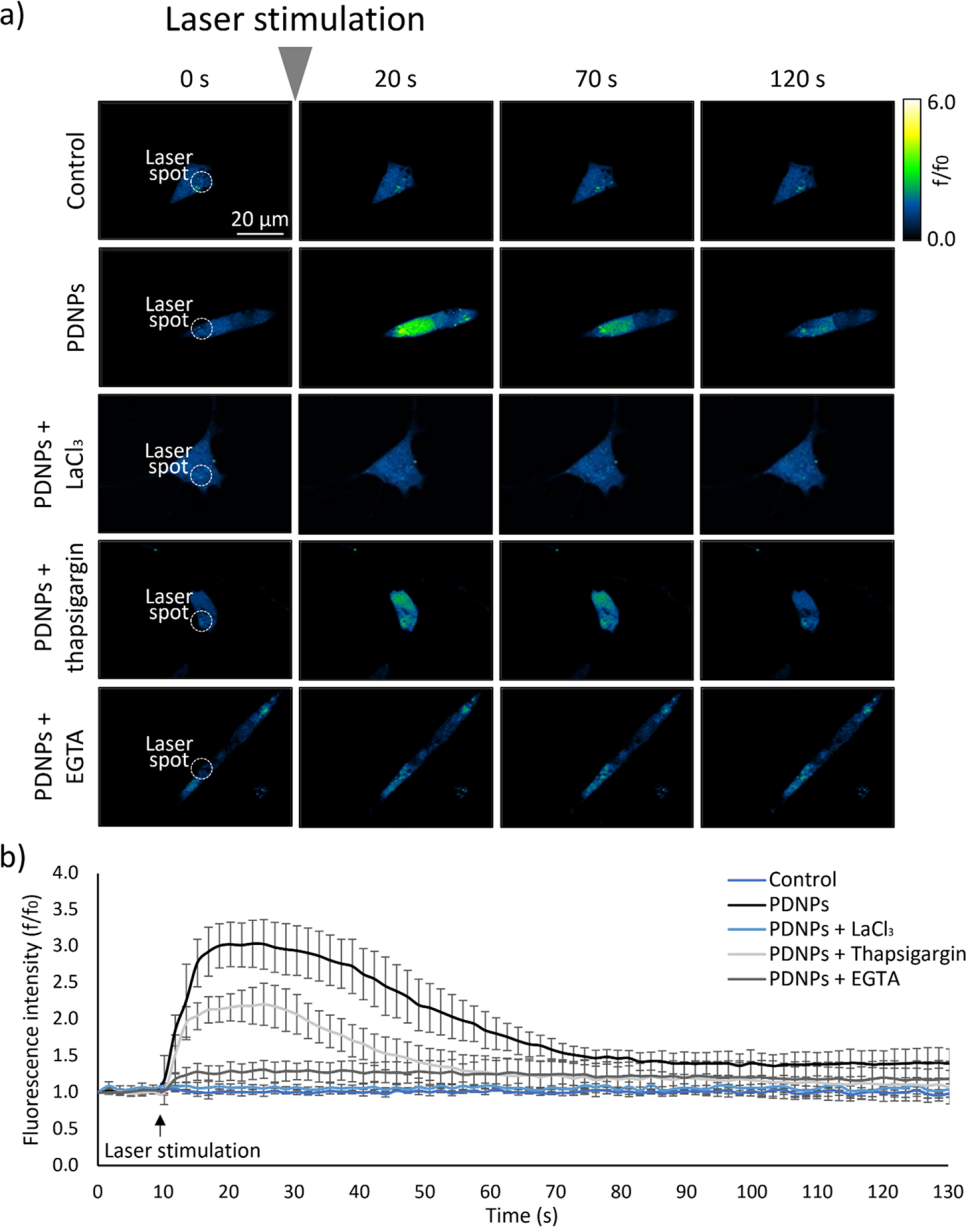
Calcium imaging on SH-SY5Y cells subjected to repeated 50 ms on/100 ms off NIR laser stimulation, for 20 times. (a) Representative time frames images. (b) Time course of the variation of cell fluorescence levels, indicative of calcium concentration (*n* = 10).

We then explored the possibility of triggering calcium transients in differentiated SH-SY5Y cells by performing a stimulation pattern of 50 ms laser on, 100 ms laser off, and 20 times (Figure 4a). Also in this case, control cells did not exhibit significant changes in fluorescence levels (Figure 4b); however, in the presence of PDNPs, we observed the production of a robust calcium transient, resulting in a fluorescence increment of 203.4 ± 32.2%.

Investigations on calcium channel involvement showed no fluorescence increment in the presence of LaCl_3_, while assessment of the Ca^2+^ sources (Figure 4b) revealed that repeated NIR laser stimulation in Ca^2+^-free conditions with EGTA led to a maximum fluorescence increase of 31.1 ± 12.2%, while in the presence of thapsigargin, this was 120.3 ± 28.7%.

As shown in the representative example reported in Figure S10, following the production of calcium transients due to the repeated NIR irradiation of individual neuron-like cells, we occasionally observed the production of transients, even in adjacent cells that were not directly irradiated. This suggests that the activation of the irradiated cells could facilitate the release of ACh necessary for the activation of an adjacent cell, thereby enabling the propagation of the calcium wave.

**Figure 5:**
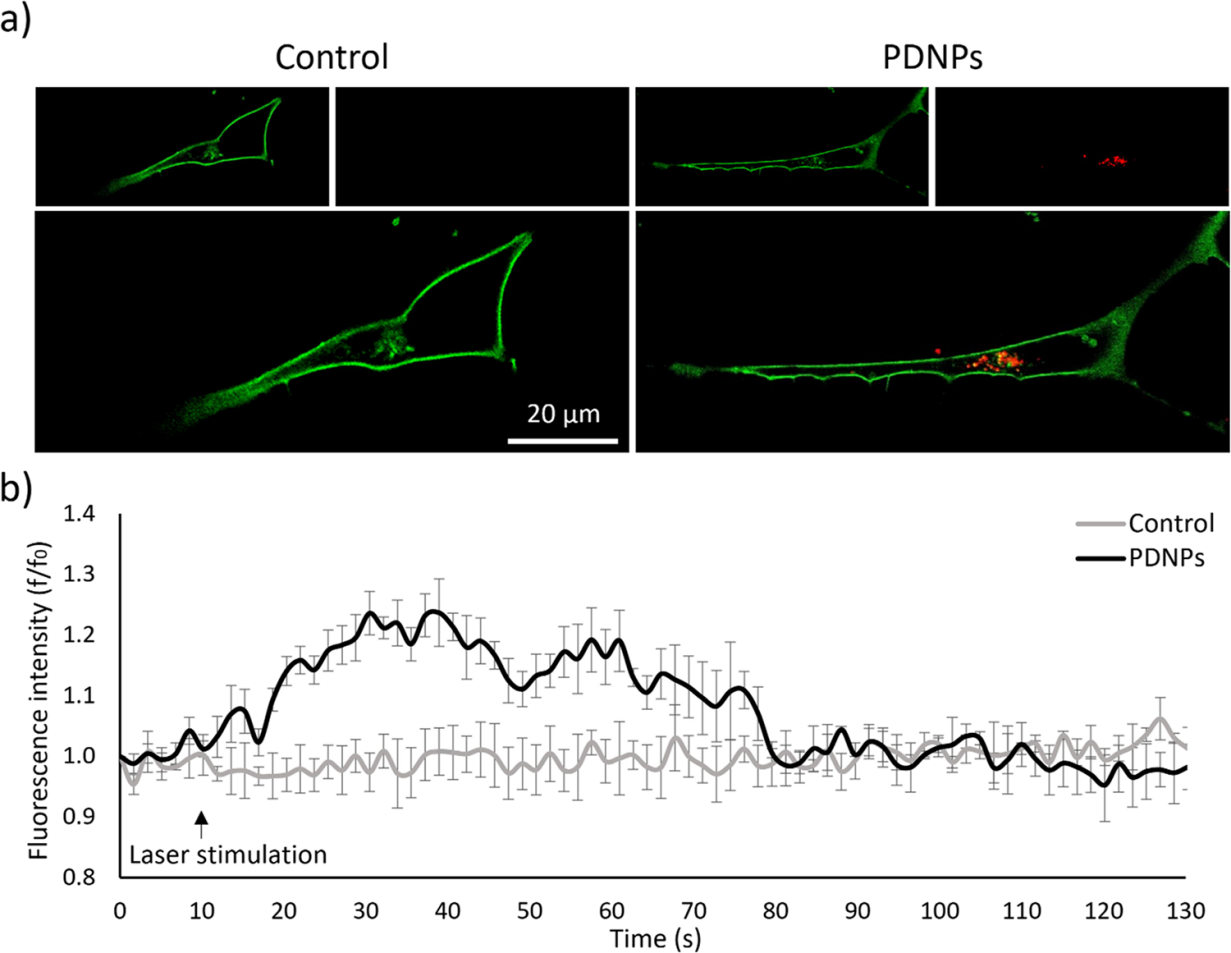
Acetylcholine release analysis in differentiated SH-SY5Y cells. (a) Representative confocal acquisitions of acetylcholine content along the plasma membrane (acetylcholine biosensor in green, DiI-PDNPs in red). (b) Time course of the variation of cell fluorescence levels, indicative of acetylcholine release, during NIR laser stimulation.

We further investigated the possibility of triggering the release of ACh upon NIR laser stimulation of cells treated with PDNPs, through the exploitation of the genetically encoded acetylcholine sensor GRAB_ACh3.0_ (Figure 5a). This sensor is based on a modified G-protein coupled receptor (GPCR) that specifically binds acetylcholine, leading to a conformational change that causes a variation in fluorescence levels depending on ACh concentration.^46^ Repeated NIR irradiation of differentiated SH-SY5Y cells did not result in an increase in fluorescence levels (Figure 5b); however, when PDNPs are present, a significant increment in cell fluorescence (23.7 ± 5.6%), indicative of ACh release, occurs.

Finally, the evaluation of dopamine levels revealed that, following treatment with PDNPs, differentiated SH-SY5Y cells exhibited a concentration of this neurotransmitter of approximately 39.7 ng/mL (*p* < 0.001), compared to the 24.2 ng/mL detected in control cells (Figure S11).

### Muscle Cell Activation upon NIR Stimulation

**Figure 6:**
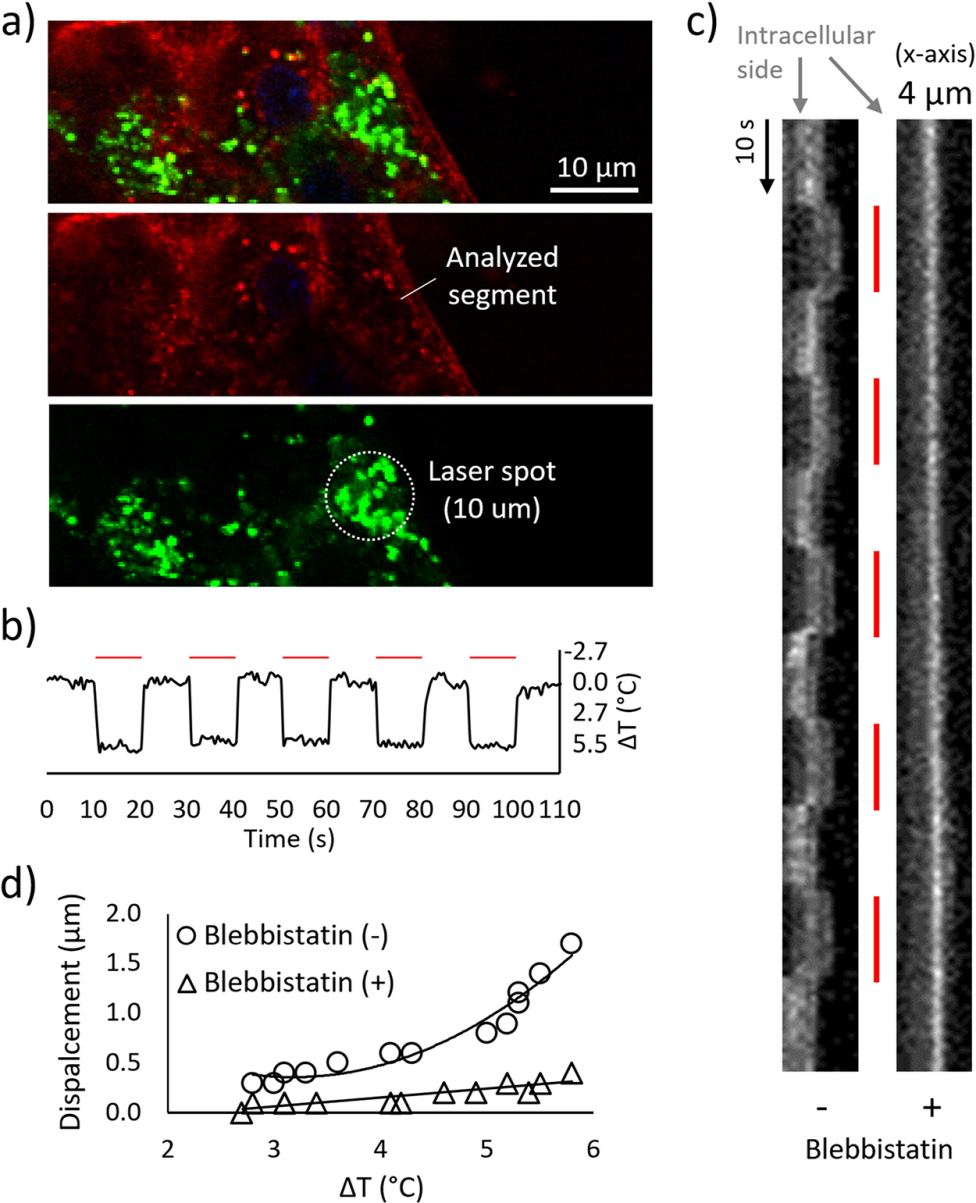
Myotube contraction analysis. (a) Representative images of C2C12 myotubes treated with PDNPs (cytoplasm in red, DiO-PDNPs in green). (b) Temperature increments provided by 10 s NIR laser stimulation in the presence of PDNPs (red dashes representing laser on). (c) Kymograph related to the analyzed segment, as shown in a), in the absence or presence of blebbistatin (red dashes representing laser on). (d) Quantitative analysis of the displacement induced by the NIR + PDNP treatment.

The ability of PDNPs to induce C2C12 myotube contraction upon NIR laser irradiation was evaluated through confocal microscopy (Figure 6a). A 10 s laser on/10 s laser off NIR stimulation, repeated 5 times, showed a reversible temperature increment (Figure 6b) that induced contractions of the myotube at subcellular level (Video S1), an observation supported by the representative kymograph reported in Figure 6c. Upon the addition of blebbistatin, an inhibitor of myosin ATPase activity, the myotube displacements were negligible, suggesting that the contractions previously observed in the absence of the inhibitor were indeed due to the actin/myosin interaction. The degree of displacement was found dependent on the temperature increment, showing a correlation that follows an exponential curve (Figure 6d) consistent with the Arrhenius relation suggested by Ferdinandus et al.^47^

### Oxidative Stress Level Increment Prevention

The effects of PDNPs on preventing oxidative stress following intracellular temperature increments were assessed using gold nanoshells (AuNSs) as a positive control of the rise in ROS levels, since gold-based nanomaterials can reach similar temperatures following NIR laser stimulation.^48,49^ AuNSs presented an average diameter of 138.7 ± 15.9 nm (Figure S12a), a *D*_h_ and a PDI, respectively, of 210.3 ± 4.1 nm and 0.10 ± 0.04 (Figure S12b), a ζ-potential of −9.6 ± 1.5 mV (Figure S12c), and a characteristic absorbance peak at 808 nm (Figure S12d). The analysis of the photothermal conversion properties of the two nanostructures revealed that the heating effects after 5 min of NIR laser irradiation produced by an aqueous dispersion of PDNPs at 100 μg/mL is equivalent to that generated by 50 μg/mL of AuNSs, resulting in both cases in a temperature increase of approximately 9 °C (Figures S12e and S12f).

**Figure 7:**
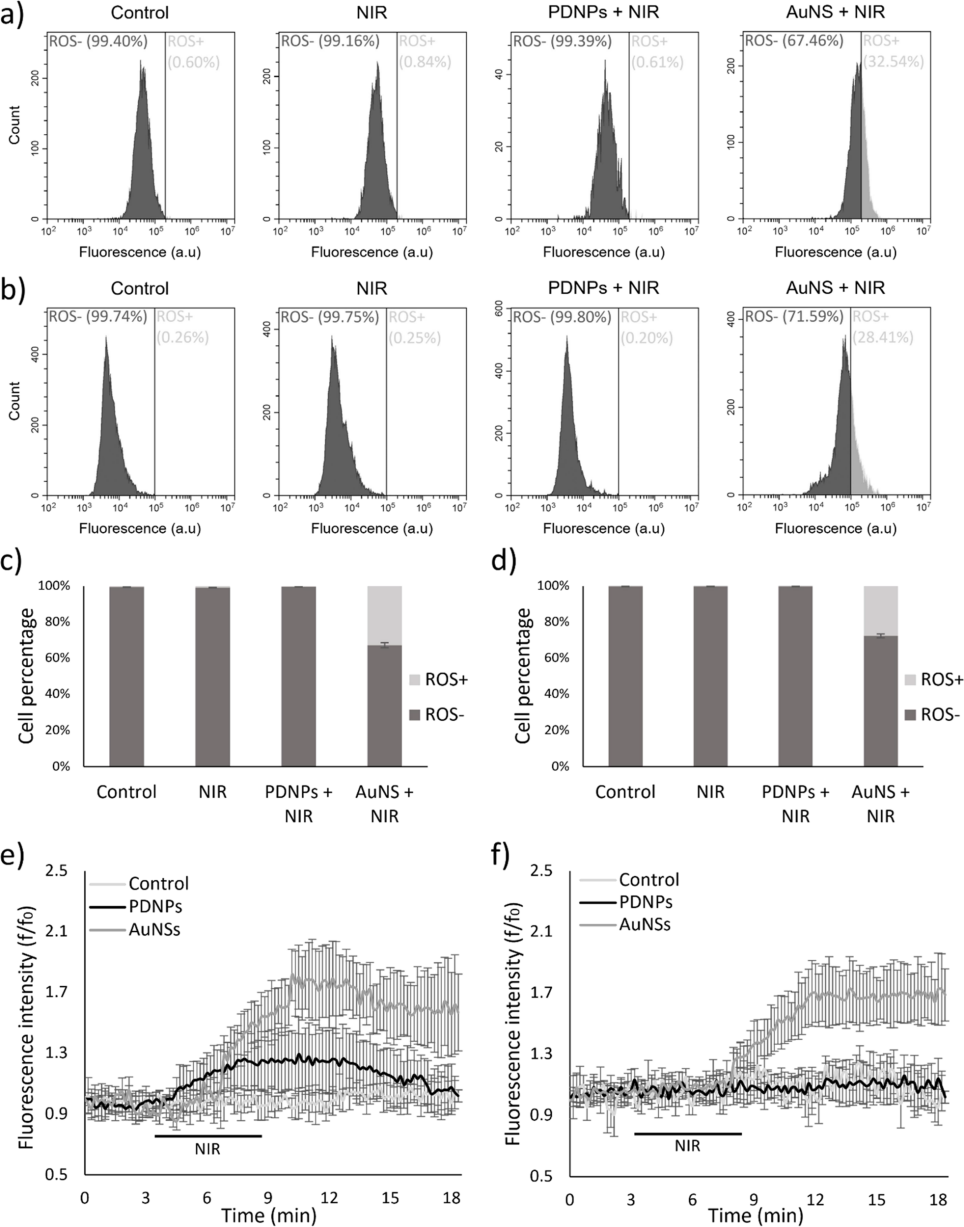
Oxidative stress level analyses of differentiated neurons and myotubes exposed to NIR irradiation. Representative flow cytometry plots of (a) differentiated SH-SY5Y and (b) differentiated C2C12 cells (in dark gray ROS-negative cells, in light gray ROS-positive cells). Oxidative stress level quantification for (c) differentiated SH-SY5Y and (d) differentiated C2C12 cells (in dark gray ROS-negative cells, in light gray ROS-positive cells; *n* = 3, ***p < 0.001). Time-course variation of fluorescence levels indicative of intracellular ROS of (e) differentiated SH-SY5Y and (f) differentiated C2C12 cells (*n* = 10).

The CellROX assay was performed, thus involving a cell-permeable reagent that upon oxidation emits strong fluorescence (Figure 7a,b). As shown in Figure 7c,d, for both neuron-like cells and myotubes, NIR laser stimulation in the absence of PDNPs did not produce any change in the percentage of ROS-positive cells. NIR irradiation of AuNSs resulted in a statistically significant increase in ROS-positive cells (32.8 ± 1.4% in neuron-like cells, 27.7 ± 1.1% in myotubes; in both cases *p* < 0.001). Conversely, the temperature increment following NIR + PDNP treatment did not show any statistically significant increase in ROS levels in both investigated cell lines.

To investigate the temporal dynamics of oxidative stress following NIR stimulation, we performed time-lapse confocal microscopy using an ROS-sensitive fluorescent dye. In neuron-like cells, NIR laser stimulation in the absence of PDNPs did not induce any significant change in fluorescence levels (Figure 7e). However, in the presence of PDNPs, fluorescence increased by approximately 29.3% upon NIR stimulation and returned to baseline within 10 min after the laser stimulus ended. NIR irradiation of AuNSs, conversely, induced an increase in ROS levels exceeding 55%, which persisted even 10 min after the laser stimulus ended.

In myotubes, NIR laser stimulation did not induce any significant increase in oxidative stress levels, in the absence or presence of PDNPs (Figure 7f). In contrast, when AuNSs were present, ROS levels increased by more than 64%, and remained sustained even 10 min after the laser stimulus ended.

### Proteomic Analysis Following Stimulation

Proteomic analysis was performed to investigate the protein pathways affected by PDNP treatment, NIR laser stimulation, and their combination (Figure S13). In neuron-like cells, PDNP treatment (PDNPs vs control) alone had a more pronounced effect than laser stimulation alone (control vs NIR), leading to a statistically significant change in the expression of 97 proteins compared to 50 proteins in the laser-only condition. Notably, the combination of PDNP treatment and NIR laser stimulation (NIR + PDNPs vs control) resulted in a significant alteration in the expression of 140 proteins. In myotubes, PDNP treatment (PDNPs vs control) was the primary driver of proteomic changes, with 98 proteins exhibiting statistically significant expression differences. In contrast, NIR laser stimulation alone (control vs NIR) led to significant alterations in only four proteins. The combined treatment (NIR + PDNPs vs control) induced a proteomic response comparable to PDNP treatment alone, with 96 proteins showing significant expression changes. As further highlighted by the clustering observed in the principal component analysis (PCA, Figure S14), laser stimulation alone appeared to have an effect just in SH-SY5Y cells. Conversely, in both cell lines, PDNP treatment alone had the most pronounced impact on protein expression modulation.

### Ex Vivo Calcium Imaging

**Figure 8:**
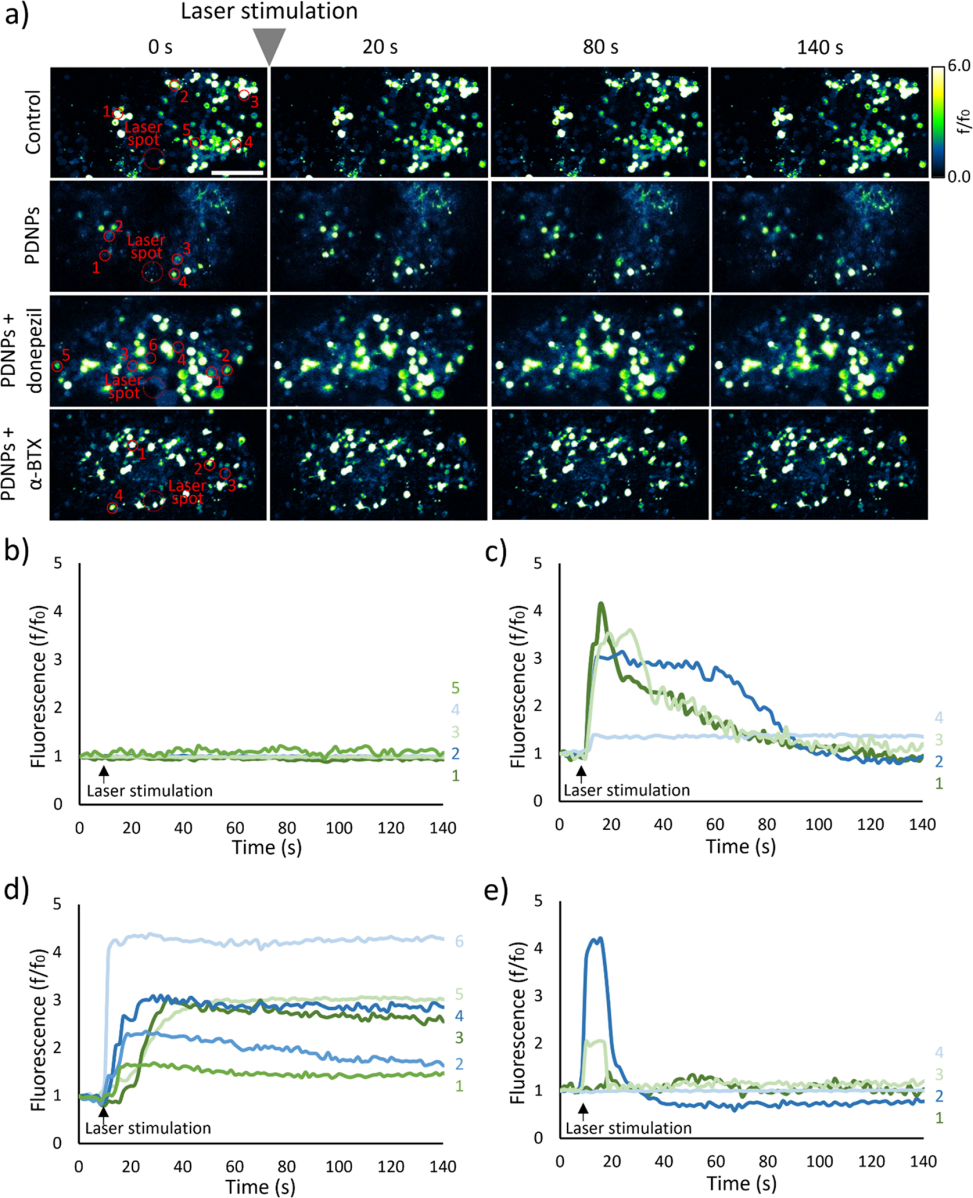
Analysis following *Drosophila* brain stimulation. (a) Representative time frames acquisition of calcium imaging performed on *Drosophila* brains (scale bar 30 μm); ROIs selected for the analysis are highlighted in red. Time course of the variation of cell fluorescence levels, indicative of calcium concentration: (b) control, (c) PDNPs treatment, (d) PDNPs + donepezil (100 nM) treatment, and (e) PDNPs + α-BTX (5 μM) treatment.

To investigate whether PDNP irradiation can manipulate fly neuronal activity, the intracellular dynamic of Ca^2+^ was studied in *Drosophila* brains expressing the fluorescent calcium indicator jGCaMP7c. Representative time frames of individual ex vivo brains during exposure to NIR laser irradiation and the related time course of the variation of cell fluorescence levels are reported in Figure 8a–e, while Figure S15 summarizes the results obtained from all the *Drosophila* brains considered in this study. As expected, without PDNPs, no significant change in fluorescence levels was detected, indicating no increase in intracellular Ca^2+^ levels. Conversely, stimulation of PDNP-treated *Drosophila* brains induced the production of strong calcium transients, as suggested by a fluorescence increment of 565.9 ± 172.3%.

NIR stimulation on PDNP-treated brains was also conducted in the presence of 100 nM donepezil, an inhibitor of the acetylcholinesterase (AChE). AChE is the primary cholinesterase in the body, responsible for catalyzing the breakdown of acetylcholine, mainly produced in neuromuscular junctions and in chemical synapses where its activity serves to terminate synaptic transmission.^50,51^ NIR stimulation in the presence of donepezil caused a significant increase in calcium influx into the irradiated cells, as indicated by a fluorescence increment of 628.4 ± 253.8%. It is worth highlighting that in the presence of the AChE inhibitor, we did not observe a calcium transient as in the previous analysis, yet a stable increment of intracellular Ca^2+^ levels. The absence of a transient event suggests that NIR + PDNP stimulation indeed promotes ACh release in *Drosophila* brains. Being donepezil present, synaptic acetylcholine levels remain constant over time, resulting in the nontransient signal herein observed.

α-bungarotoxin (α-BTX) is a neurotoxin known to irreversibly inhibit synaptic acetylcholine release by specifically binding to nicotinic acetylcholine receptors (nAChR), which are transmembrane ion channel abundantly expressed in the central nervous system of *Drosophila*.^52−55^ NIR stimulation in the presence of α-BTX resulted in a temporary increase of the fluorescence levels (53.0 ± 32.4%) due to the production of calcium transients, which was, however, significantly smaller in amplitude with respect to the tests carried out in the absence of the toxin.

## Discussion

Several in vitro studies have proven the possibility of remotely modulating cellular activity by using inorganic nanostructures. For instance, Hung et al. showed the activation of neuronal cells by applying a radio frequency electromagnetic field to superparamagnetic manganese ferrite (MnFe_2_O_4_) nanoparticles.^56^ Moreover, Nakatsuji et al. were able to modulate neuronal activity using surface-engineered plasmonic nanorods (pm-AuNRs).^57^ Additionally, the employment of gold nanoshells studied by Marino et al. is interesting for performing remote myotube activation.^35^ As a final example, Soloviev et al. demonstrated the ability to remotely control the opening of potassium channels in the smooth muscles of a rat thoracic aorta using 5 nm diameter plasmonic gold nanoparticles.^58^ However, the application of these and analogous approaches is limited by the inorganic nature of the nanostructures, due to the concerns related to their biodegradability or to the extended retention of the material within the body.^59^

In this study, by using fully organic, biocompatible, and biodegradable nanoparticles, we effectively demonstrated the precise modulation of single-cell activity.

According to previous findings from our group,^60^ PDNPs have a spherical shape, a diameter of approximately 200 nm, a monodisperse distribution, and a strongly electronegative ζ-potential. The nanoparticles proved to be stable for several days in the culture medium used for biological experiments, while they were found to be affected in terms of degradability by lysosomal enzymes. By comparing the free radical scavenging activity of PDNPs with that of Trolox, a water-soluble vitamin E analogue, we confirmed the high antioxidant capacity of the nanoparticles, a crucial property for preventing the increase of free radicals caused by intracellular heating.^61^

For both types of cells used in this study, that is, neuron-like cells and myotubes, the cell-nanoparticle interactions were initially assessed by focusing on the PDNP impact on cell viability. As expected, PDNPs showed a satisfactory biocompatibility profile; further experiments indicated that the nanoparticles were predominantly localized within lysosomes, with a higher content internalized by myotubes with respect to neuron-like cells. We also assessed the effect of NIR laser stimulation on cells treated with PDNPs, applying stimulation for a significantly longer duration than that one exploited for the cellular activation experiments. No statistically significant impact on cell viability was observed at the concentration used in our study, further supporting the safety of the applied treatment conditions.

The ability of PDNPs to promote cell differentiation was evaluated, and in the case of myotubes, no statistically significant differences compared to the control cells were found. Conversely, treating neuron-like cells with PDNPs significantly promoted neuronal differentiation, with approximately a doubling in the median length of neurites and in the median number of neurites per cell.

The differential effect of PDNPs on SH-SY5Y and C2C12 differentiation is likely due to the distinct roles of oxidative stress in neurogenesis and myogenesis. In neuronal differentiation, oxidative stress plays a crucial role in modulating signaling pathways such as MAPK/ERK and NGF, where excessive ROS levels can impair neurite outgrowth.^62^ By reduction of oxidative stress, PDNPs create a more favorable redox environment that promotes neuronal differentiation. Conversely, myogenic differentiation is regulated primarily by transcription factors like MyoD and myogenin, which respond to mechanical and metabolic cues rather than to redox balance.^63^ This could give a hint about why PDNP treatment enhances SH-SY5Y differentiation but does not significantly impact C2C12 myotube formation; indeed, the ability of PDNPs to shift neuronal cells toward a more favorable state for differentiation could open alternative and interesting perspectives for neuronal regeneration applications.^64^

As previously mentioned, our goal was to use a fully organic and biodegradable nanostructure to achieve remote thermal activation of cells; due to the difference in the internalization extents between the two cell lines, we used different laser power levels to achieve temperatures suitable for nondisruptive cell activation.^44^ We observed that PDNPs could be repeatedly irradiated with an NIR laser source, each time resulting in a fully reversible temperature increase confirmed by a step-like change in fluorescence, and without any drop in performance over time. The ability to produce a reversible effect allows for exerting precise control over cellular activity: once the stimulus is removed, the effect indeed also ceases, returning to a physiological status.

We leveraged the photothermal conversion capabilities of polydopamine,^65^ by working at a 100 μg/mL concentration and adjusting the laser power to achieve an intracellular temperature over 41 °C.

In the case of neurons, reaching a temperature between 41 and 42 °C is considered a threshold for activating cation channels of the transient receptor potential (TRP) family, particularly the TRP subfamily V member 1 (TRPV1) channels, which are thermosensitive channels^66,67^ responsible for the production of cationic influx, such as Ca^2+^ influx, and the subsequent neuronal activation.^66,68^ Herein, we investigated the actual activation of neuron-like cells by verifying the production of Ca^2+^ transients. Calcium is a universal second messenger that regulates key activities in all eukaryotic cells and is particularly important for neurons, as it plays a role in transmitting depolarizing signals and contributing to synaptic activity. Consequently, neurons have developed extensive and intricate calcium signaling pathways to link calcium influx with their biochemical processes.^69^ Initially, we individually stimulated the cells with a single laser irradiation of 50 ms, and as expected, we observed no production of calcium transients in the absence of nanoparticles. Conversely, when NIR laser stimulation was performed in the presence of PDNPs, an immediate generation of a calcium transient occurred, albeit of low amplitude. Considering that TRP channels are predominantly present on the plasma membrane,^70^ it is likely that the influx of extracellular Ca^2+^ is responsible for generating the observed transients. To validate this, we repeated the stimulation under Ca^2+^-free conditions, resulting in a complete absence of transients, thus confirming the involvement of extracellular Ca^2+^ influx. However, when we depleted Ca^2+^ storage within the ER, we observed the production of a Ca^2+^ transient similar to that obtained in normal calcium conditions, confirming the contribution of the extracellular calcium source to the Ca^2+^ transient production. The involvement of calcium channels in the transients observed was further confirmed using LaCl_3_, a known calcium channel blocker.^71^ In the presence of LaCl_3_, no calcium transients were observed following laser stimulation, confirming the hypothesis that calcium channels play a crucial role in the photothermal effect produced by PDNPs.

Continuing, we then proceeded by following a pattern of 50 ms repeated laser pulses, and even with this irradiation pattern, in the absence of nanoparticles, no fluorescence variation was observed, indicating no calcium transient production. However, in the presence of PDNPs, a high-amplitude Ca^2+^ transient was observed, significantly more intense than that one observed with a single 50 ms stimulation. Also in this scenario, we investigated the source of calcium responsible for transient flow production. After depleting Ca^2+^ stored within the ER, we once again observed the generation of a high-amplitude transient; however, under Ca^2+^-free conditions, we observed the production of a low-amplitude transient. This result indicates that following a repeated thermal stimulation, the ER minimally contributes to the increase in cytoplasmic Ca^2+^ concentration. The impact of calcium release from the ER store remains poorly understood, though some studies suggest its potential significance: for example, Venkiteswaran et al. demonstrated that Ca^2+^ release from the ER in neurons is essential in *Drosophila*.^72^ Interestingly, we observed that some of the high-amplitude Ca^2+^ transients occurred in cells not directly interested in NIR stimulation, suggesting a possible mechanism whereby an activated neuron transmits excitation to the adjacent cell. In this regard, it is interesting to point out that calcium waves are known to play a crucial role in fostering neural network maturation, particularly by regulating neurite outgrowth.^73^

The activation of individually stimulated neuron-like cells was ultimately successfully confirmed through the use of a plasmid encoding the ACh sensor named GRAB_ACh3.0_. By producing this biosensor on the plasma membrane of transfected neuron-like cells, we were able to monitor ACh release in real-time through the resulting changes in fluorescence levels depending on the neurotransmitter concentration. We observed that only in cells treated with PDNPs, the repeated NIR laser stimulation was able to induce the release of the neurotransmitter. This result further corroborates what was previously observed: thanks to a precise and adjustable thermal stimulus achieved through NIR + PDNP stimulation, we can generate a calcium wave that can propagate to adjacent cells through the release of ACh. To the best of our knowledge, this is the first example of using fully organic nanostructures to precisely trigger ACh release, and this result holds significant potential for enhancing the understanding of neural circuits and synaptic plasticity, developing alternative treatments for neurological disorders, and fostering nonconventional bioengineering solutions.

Our findings indicate that PDNPs significantly increase dopamine levels in treated neuron-like cultures. This effect could be attributed to the intrinsic structural and chemical similarity between polydopamine and endogenous dopamine, which may influence cellular dopamine metabolism.^74^ One possible mechanism involves the interaction of PDNPs with enzymes responsible for dopamine synthesis, storage, or degradation, potentially leading to an accumulation of the neurotransmitter. Additionally, the PDNP antioxidant properties might contribute to dopamine preservation by reducing oxidative degradation. Further studies are required to elucidate the molecular pathways involved and determine whether this phenomenon can be leveraged for neuroprotective or neuromodulatory applications.

In the case of myoblasts, it is well-known that an increment of the intracellular temperature above 37 °C raises the probability of triggering muscle contractions, by approaching nearly 100% with a temperature increase of about 5–6 °C (starting from 37 °C).^75^ For instance, febrile seizures in young children occur when their body temperature exceeds 38 °C.^76^ These contractions result from the formation of cross-bridges between actin and myosin, facilitated by the rise in temperature, a mechanism completely distinct from that one triggered by electrical stimulations.^77^ We observed that the NIR + PDNP stimulation leads to the generation of reversible contraction at the subcellular level; the cell displacement caused by the contraction was confined to the area of irradiation, and a correlation between achieved temperature and degree of displacement was found. When blebbistatin,^78^ a myosin ATPase inhibitor was added, no myotube contraction was observed, suggesting that the displacements were due to thermodynamic changes in the interaction between myosin and the actin-tropomyosin-troponin complexes.

We then evaluated whether PDNP irradiation and the subsequent rise in intracellular temperature could, as known, elevate oxidative stress levels,^79−81^ observing that, conversely to other nanostructures used for photothermal stimulation,^82^ PDNPs successfully prevented the rise in oxidative stress levels due to their high antioxidant properties. We also investigated the dynamic interaction between the photothermal effects induced by PDNPs and their antioxidant properties through a real-time acquisition. Our analysis aimed to determine whether, following the onset of laser stimulation, an initial increase in ROS levels triggered by the photothermal heat occurred, followed by a reduction due to the antioxidant activity of PDNPs, or whether PDNPs could prevent the rise in oxidative stress levels altogether. Notably, at the neuronal level, we observed an initial increase in intracellular ROS, followed by a decrease after laser stimulation ceased, indicating a sequential process in these cells. Conversely, in myotubes, no significant rise in ROS levels was observed following laser stimulation, suggesting protective action by PDNPs. The observed differences could be attributed to variations in cellular response mechanisms and oxidative stress control. For instance, myotubes, being specialized to endure high metabolic stress during muscle contractions and physical exertion, may exhibit greater resilience to oxidative damage.^83^ Additionally, as previously noted, there is a substantial difference in the ability of the two cell types to internalize PDNPs, which could also significantly contribute to the observed differences.

To further investigate the molecular mechanisms underlying the phenomena observed, we conducted a comprehensive proteomic analysis. This approach allowed us to gain insight into how treatment with PDNPs and laser stimulation may affect cellular pathways at the molecular level.

In neuron-like cells, the comparison between control cells and NIR-irradiated control cells (control vs NIR) revealed enrichment of Gene Ontology (GO) terms such as *response to heat* and *protein refolding*, indicating a cellular response to thermal stress. As expected, this resulted in the upregulation of Heat Shock Protein 72 (HSP72), a molecular chaperone involved in protecting the proteome from stress.^84^ The comparison between PDNP-treated cells and control cells (PDNPs vs control) led to the enrichment of multiple GO terms related to neuronal development and function, including *axon guidance*, *brain development*, *nervous system development*, *regulation of calcium ion transmembrane transport*, and *neurite outgrowth*. Several proteins showed statistically significant differential expression following PDNP treatment, and among the most upregulated, we found SRBS2, which plays a key role in regulating synaptic transmission in the mammalian brain,^85^ ANF, involved in fluid and electrolyte balance regulation in both the central and peripheral nervous system,^86^ and PLMN, which contributes to neuronal repair and neurite growth.^87^ Similarly, the comparison between NIR + PDNP-treated cells and control cells (NIR + PDNPs vs control) resulted in the enrichment of several GO terms and significant changes in protein expression. Due to the presence of laser stimulation, we observed again the enrichment of GO terms such as *response to heat* and *protein refolding*, along with the overexpression of AUXI, a member of the DNAJ/HSP40 protein family that modulates molecular chaperone activity in response to stress and that is also involved in synaptic development.^88^ Notably, this comparison shared several enriched GO terms with the PDNPs vs control condition, including *brain development*, *regulation of neuron migration*, *synapse organization*, *dendrite development*, *central nervous system development*, and *regulation of neurotransmitter secretion*. Consequently, a large number of proteins showing significant expression changes in the PDNPs vs control comparison were also differentially expressed here. Among them, SRBS2 was again upregulated, alongside ELFN1, a postsynaptic protein crucial for synaptic development,^89^ MTAP2, a well-known neuronal marker essential for microtubule assembly, a key step in neurogenesis,^90^ and SYN1, which plays a role in modulating neurotransmitter release at synapses.^91^

As previously observed, in the case of myotubes, the predominant effect of PDNP treatment on protein expression modulation is even more pronounced than that observed in neuron-like cells. The comparison between PDNP-treated cells and control cells (PDNPs vs Control) led to the enrichment of several GO terms related to muscle development and function, such as *muscle organ development*, *cardiac myofibril*, *muscle cell cellular homeostasis*, *striated muscle cell differentiation*, *M band*, *Z disc*, and *response to steroid hormone*. Among the many differentially expressed proteins that contributed to this enrichment, some particularly noteworthy examples include CO4A2, a type IV collagen subunit and a key component of the basal membrane in muscle fibers, which is involved in structural stability and extracellular matrix–muscle cell communication,^92^ MGP, the muscle isoform of glycogen phosphorylase, which plays a role in regulating myostatin expression during muscle development,^93^ and TFR1, a transmembrane receptor essential for iron uptake, a critical element for mitochondrial function in muscle cells and the energy production required for muscle contraction.^94^ Similarly, the comparison between PDNP-treated and NIR-stimulated cells versus control cells (NIR + PDNPs vs control) also resulted in the enrichment of a large number of GO terms, most of which were related to muscle cells. Notably, over 60% of the differentially expressed proteins overlapped with those identified in the PDNPs vs control comparison, confirming that, particularly in myotubes, protein expression was predominantly modulated by the PDNP treatment alone. The enriched GO terms in this condition included *muscle cell development*, *regulation of striated muscle tissue development*, *contractile muscle fiber*, and an *insulin-like growth factor receptor signaling pathway*. Among the most significantly differentially expressed proteins, we found CREG1, a cytoplasmic protein involved in muscle differentiation and homeostasis,^95,96^ LYOX and LOXL1, lysyl oxidases that play a role in extracellular matrix organization and response to steroid hormones,^97,98^ and eventually IBP5, an insulin-like growth factor binding protein that regulates muscle growth and smooth muscle cell differentiation.^99^

Considering that the cells were exposed to NIR laser stimulation for a significantly longer duration than the one used for cellular activation, exclusively to highlight the potential effects of photothermal conversion, these findings confirm that the predominant effect on the proteome was induced by the PDNP treatment. Moreover, obtained results indicate that the nanoparticles used in this study can not only remotely control cellular activation but also play a role in promoting neurogenesis, synapse organization, and neurotransmitter regulation, suggesting that PDNPs may contribute to neuronal plasticity and functional maturation. Similarly, the upregulation of key proteins involved in muscle differentiation, extracellular matrix remodeling, and metabolic homeostasis supports the idea that PDNPs could play a role in improving muscle growth and functional integrity. These findings suggest important perspectives for the potential application of PDNPs as bioactive nanomaterials capable of influencing cellular development and functional properties across different tissue types.

Eventually, the ability to precisely control neuronal cell activity was evaluated ex vivo in dissected *Drosophila melanogaster* brains by exploiting calcium imaging performed thanks to genetically encoded calcium indicators (GECI), such as jGCaMP7c, commonly used to track the activity of large populations of neuronal cell bodies.^100^ After the optic lobe area was irradiated without PDNPs, no changes in fluorescence levels were observed, indicating no calcium influx. However, in brains pretreated with PDNPs, laser stimulation resulted in multiple neuronal activations marked by the production of various calcium transients. Moreover, we observed increases in intracellular Ca^2+^ levels even in neurons located far from the irradiation spot, suggesting that the production of calcium transients led to the release of ACh by the irradiated neurons, which in turn activated neurons distant from the irradiation spot. To confirm this, we conducted experiments in the presence of donepezil, an AChE inhibitor, to prevent the degradation of ACh after its release, thus producing sustained signals. Following NIR laser stimulation, we indeed observed a prolonged increase in the fluorescence levels of several neurons, unlike the temporary spikes observed without the AChE inhibitor. For further confirmation, we provided PDNP-pretreated brains with α-BTX, an ACh receptor inhibitor: in this case, calcium transients were evoked, but significantly attenuated in terms of amplitude, similar to the findings of Nakamura et al.^101^ These findings suggest that by irradiating PDNPs, we can activate neurons even in a complex environment like the brain; moreover, by inhibiting the binding of ACh to its receptors, the generation of a stronger signal capable of reaching areas of the brain distant from the irradiated regions cannot be achieved.

The ex vivo studies on *Drosophila melanogaster* brains investigated the effects of PDNP-mediated photothermal stimulation in a structured neural environment, which is widely used for studying neurobiological processes. The excellent biocompatibility, biodegradability, and strong NIR absorption of PDNPs support their potential translation to mammalian systems. While challenges such as biodistribution and NIR penetration depth remain, these can be addressed through optimization strategies, including targeted functionalization and fiber-optic-assisted light delivery.^102−104^

## Conclusions

We demonstrated the thermal control of single neuron-like cells or myotubes by using PDNPs as photothermal transducers, an approach allowing for the targeted modulation of cellular activity with high spatiotemporal resolution, leveraging the unique photothermal properties of the nanoparticles. Our findings suggest that PDNPs could be utilized in a wide range of biomedical applications, from developing alternative therapeutic strategies for neurological and muscular disorders to advancing research in cellular physiology and bioengineering. Their biocompatible and biodegradable nature further enhances their potential for clinical translation, ensuring minimal adverse effects. Overall, our study underscores the vast potential of NIR laser-activated nanoparticles in both research and therapeutic contexts, paving the way for innovative solutions in the modulation of cellular functions.

## Methods

### Nanoparticle Synthesis and Characterization

PDNPs were prepared following a procedure outlined in a previous work of our group.^105^ In summary, a mixture of 90 mL of Milli-Q water, 40 mL of ethanol, and 2 mL of an ammonium hydroxide solution (Sigma) was gently stirred at room temperature (RT) for 30 min. Subsequently, 10 mL of Milli-Q water containing 0.5 g of dopamine hydrochloride (Sigma) was added. The reaction mixture was stirred for 24 h, and the resulting suspension was diluted 1:1 in ethanol before being centrifuged at 8960*g* for 30 min at 4 °C. The obtained nanoparticle pellet was then resuspended in Milli-Q water following three washing steps at 12,000*g*. The concentration of PDNPs was determined by weighing freeze-dried samples.

For internalization and intracellular localization examination, PDNPs were labeled with either DiO or DiI (Vybrant Multicolor Cell-Labeling Kit, Thermo Fisher Scientific). In both cases, 10 μM dye was added to 1 mL of Milli-Q water containing 5 mg of nanoparticles, and the mixture was stirred for 2 h. The labeled nanoparticles were subsequently washed three times with Milli-Q water via centrifugation at 12,000*g*.

PDNP size and morphology were evaluated through SEM and TEM, followed by analysis using Gwyddion software. For SEM imaging, 10 μL of a 100 μg/mL nanoparticle dispersion was allowed to dry on a silicon substrate, followed by gold sputtering using a Quorum Tech Q150RES Gold Sputter Coater (30 mA, 60 s). Imaging was conducted using a Helios NanoLab 600i FIB/SEM Dual-Beam SEM system (FEI). For TEM imaging, 10 μL of the sample suspension were placed onto a 150 mesh copper grid coated with a thin layer of carbon, and bright-field TEM images were obtained using a JEOL JEM-1400Plus TEM with a thermionic source (LaB_6_) operating at 120 kV.

DLS measurements were performed by using a Malvern Zetasizer Nano ZS90 instrument to determine the *D*_h_, PDI, and ζ-potential. *D*_h_ and PDI values were evaluated by using a 100 μg/mL dispersion in polystyrene cuvettes, while ζ-potential measurements were conducted on the same dispersions placed within folded capillary cells (Malvern Zetasizer Nano series).

A stability assay in cell culture medium was also performed on 100 μg/mL PDNP dispersions. We used high-glucose Dulbecco’s modified Eagle’s medium (DMEM, Gibco), supplemented with 10% heat-inactivated fetal bovine serum (FBS, Gibco), 1% l-glutamine (stock 200 mM, Gibco), and 1% penicillin-streptomycin (100 IU/mL of penicillin and 100 μg/mL of streptomycin, Gibco). *D*_h_ and PDI values were analyzed over 1 h with single acquisitions every 12 min and over 6 days with acquisitions every 24 h.

To evaluate the effects of a lysosomal-mimicking environment, PDNPs were incubated at 37 °C in a solution of the lysosomal protease cathepsin B (0.6 IU/mL), reaching a final concentration of 100 μg/mL. Variations of *D*_h_ and PDI values were analyzed over 1 h with single acquisitions every 12 min and over 6 days with acquisitions every 24 h.

The absorbance between 600 and 1000 nm of aqueous dispersions of 100 μg/mL PDNPs was acquired with a PerkinElmer UV/vis spectrophotometer (Lambda 45).

The antioxidant capacity of PDNPs was evaluated using the Total Antioxidant Capacity Assay Kit (Sigma). A 100 μL portion of a 10 μg/mL PDNP dispersion in Milli-Q water was combined with 100 μL of Cu^2+^ working solution and incubated at RT shielded from light for 90 min. Subsequently, the absorbance of the samples was measured at 570 nm using a Victor X3Multilabel Plate Reader (PerkinElmer). The Trolox-equivalent antioxidant activity of PDNP was determined based on a standard curve.

### Cell culture

In vitro experiments were conducted on differentiated SH-SY5Y (HTL95013, ICLC) cells and differentiated C2C12 (CRL-1772, ATCC) cells.

SH-SY5Y cells were cultured under proliferative conditions using DMEM F-12 (Gibco), supplemented with 10% heat-inactivated FBS, 1% l-glutamine (stock 200 mM), and 1% penicillin-streptomycin (100 IU/mL penicillin and 100 μg/mL of streptomycin). Following seeding, differentiation in neuron-like cells was promoted by replacing the proliferative medium with high-glucose DMEM supplemented with 1% heat-inactivated FBS, 1% l-glutamine (stock 200 mM), 1% penicillin-streptomycin (100 IU/mL of penicillin and 100 μg/mL of streptomycin), 10 μM all-trans-retinoic acid (Thermo Scientific), and 50 ng/mL of human brain-derived neurotrophic factor (hBDNF, Sigma).

C2C12 cells were cultured under proliferative conditions using high-glucose DMEM supplemented with 10% heat-inactivated FBS, 1% l-glutamine (stock 200 mM), and 1% penicillin-streptomycin (100 IU/mL penicillin and 100 μg/mL of streptomycin). Once 90% of confluence was reached, differentiation in myotubes was promoted by replacing the proliferative medium with high-glucose DMEM supplemented with 2% heat-inactivated horse serum (HS, Gibco), 1% l-glutamine (stock 200 mM), 1% penicillin-streptomycin (100 IU/mL of penicillin and 100 μg/mL of streptomycin), and 1% insulin-transferrin-sodium selenite (1 mg/mL of human recombinant insulin, 0.55 mg/mL of human transferrin, and 0.5 μg/mL of sodium selenite, Sigma).

### Nanoparticle/Cell Interaction

The impact of PDNPs on cell viability was evaluated by using the Quant-iT PicoGreen dsDNA Assay Kit (Invitrogen). Cells were seeded in a 96-well plate (Corning) at a density of 10,000 cells/cm^2^ and allowed to adhere for 24 h in proliferation medium. Thereafter, cells were switched to differentiation medium containing increasing concentrations of PDNPs (0.00, 6.25, 12.50, 100.00, 250.00, and 500.00 μg/mL) and maintained in incubation for either 24 or 72 h in the case of neuron-like cells and either 72 or 144 h in the case of myotubes. Following exposure to PDNPs, cells underwent washing with Dulbecco’s phosphate-buffered saline (DPBS, Sigma) and three cycles of freezing/thawing (from −80 to 37 °C) in 100 μL of Milli-Q water to facilitate cell lysis and dsDNA release. The PicoGreen assay was carried out by combining cell lysate, PicoGreen reagent, and TRIS-EDTA (TE) buffer in Corning Costar 96-well black polystyrene plates according to the manufacturer’s protocol. Fluorescence levels were measured using the Victor X3Multilabel Plate Reader (λ_ex_ 485 nm, λ_em_ 535 nm; PerkinElmer). The same assay was also used to evaluate the impact of laser stimulation on the cell viability. Cells were cultured and treated as described above and then subjected to 5 min of NIR stimulation using a laser source capable of irradiating multiple cells simultaneously (808 nm, 541 mW, 2.5 mm of spot diameter; Roithner Lasertechnik RLTMDL-808-500). After an additional 24 h of incubation, viability was assessed to determine any potential negative effect.

To observe the cellular uptake of PDNPs, cells were plated in CELLview dishes (VWR) at a density of 10,000 cells/cm^2^ and allowed to incubate overnight in a proliferation medium. Following incubation periods with differentiation medium containing 100 μg/mL of DiO-PDNPs (24 or 72 h in the case of neuron-like cells, 72 or 144 h in the case of myotubes), the cultures underwent washing with DPBS, fixation using 4% paraformaldehyde (PFA) in DPBS at 4 °C for 20 min, and rinsing twice with DPBS. Upon fixation, cells were incubated for 1 h at 37 °C in DPBS supplemented with 5 μg/mL of Hoechst (Invitrogen) and 2.5 μg/mL of TRITC-phalloidin (Sigma) for nucleus and f-actin staining, respectively. After the incubation, cells were washed twice with DPBS and imaged using a confocal microscope (C 2s system, Nikon) equipped with a 60× oil immersion objective.

Cellular uptake was quantitatively assessed through flow cytometry. Cells were seeded at a density of 10,000 cells/cm^2^ in 24-well plates (Corning) and incubated overnight in a proliferation medium. Following the incubation periods previously described with differentiation medium containing 100 μg/mL DiO-PDNPs, the cultures were washed with DPBS, detached from the wells, and analyzed using a CytoFLEX platform (λ_ex_ = 488 nm, λ_em_ = 525/40 nm; Beckman Coulter).

Intracellular localization of nanoparticles was examined using confocal microscopy, following mitochondria and lysosomes staining. Following the same already described culture protocols, cells were washed with DPBS and then incubated for 30 min in DPBS containing 5 μg/mL Hoechst and either 1 μM tetramethyl rhodamine methyl ester (Life Technologies) for mitochondria staining or 1 μM LysoTracker Red (Invitrogen) for lysosomes staining. After two DPBS washes, the cultures were imaged by using confocal microscopy. To assess colocalization between DiO-PDNPs and labeled organelles, the NIS-elements software (Nikon) was used to calculate the Pearson’s correlation coefficient.

### Effects on Cell Differentiation

For studying the impact of PDNPs on neuronal differentiation, SH-SY5Y cells were cultured in CELLview dishes at low cellular density (1000 cells/cm^2^). After 24 h in a proliferation medium, cells were cultured for 72 h in differentiation medium with or without 100 μg/mL of PDNPs. After 72 h, cells were fixed in 4% PFA in DPBS at 4 °C for 20 min, and an immunostaining procedure was conducted to label tubulin β-III. Cells were incubated with 10% goat serum (GS, Sigma) containing 0.3 μg/mL antitubulin β-III antibody produced in rabbit (Sigma) for 3 h and thereafter for 40 min in 10% GS containing 10 μg/mL of F(ab′)2-goat anti-Rabbit IgG (H + L) Alexa Fluor 488 conjugate (Invitrogen) and 5 μg/mL of Hoechst. Subsequently, cells were washed three times with DPBS and imaged using fluorescence microscopy (Eclipse Ti, Nikon) with a 10× objective. Acquisitions were processed and compared using ImageJ software.

To study the impact of PDNPs on myotube differentiation, C2C12 cells were cultured in CELLview dishes at a density of 10,000 cells/cm^2^. After 24 h in a proliferation medium, cells were cultured for 144 h in a differentiation medium with or without 100 μg/mL of PDNPs. Cells were then incubated in DPBS supplemented with 5 μg/mL of CellMask Orange Plasma Membrane Stain (Invitrogen) and 5 μg/mL of Hoechst and, after 15 min, washed three times with DPBS and imaged using a confocal microscope (C 2s system, Nikon) equipped with a 60× oil immersion objective. Acquisitions were processed and compared using the ImageJ software.

### PDNP Photothermal Conversion Ability

To assess the photothermal conversion ability of PDNPs, for both cell lines, a density of 10,000 cells/cm^2^ was seeded onto a glass-bottom dish and incubated for 24 h in proliferation medium. Thereafter, cells were incubated in differentiation medium or differentiation medium supplemented with 100 μg/mL of DiI-PDNPs. After 72 h of incubation in the case of neuron-like cells and after 144 h of incubation in the case of myotubes, cells underwent 30 min of incubation at 37 °C with medium supplement with 5 μg/mL of Hoechst and 1 μM Lysosomes Thermo Green (LTG), a fluorescent temperature-sensitive dye. Subsequently, samples were washed with DPBS and incubated in a medium suitable for confocal microscopy time-lapse imaging. This specific medium was composed of HEPES-supplemented (15 mM) phenol red-free DMEM (Gibco), 1% heat-inactivated FBS, 1% l-glutamine, 1% sodium pyruvate, and 1% penicillin-streptomycin. Throughout the experiment, the temperature of the culture medium was maintained at 37 °C using a microscope temperature-controlled chamber (TOKAI-HIT). Fluorescence imaging was conducted using an FV1200 confocal microscope (Olympus) equipped with an oil immersion objective lens (PLAPON 60×, NA = 1.42, Olympus). The FV10-ASW 4.2 software (Olympus) was employed to control the camera and filters and to record data. During observations, an NIR laser (808 nm wavelength, 10 μm spot diameter, 100 mW, FiberLabs Inc.) was employed to irradiate single cells through the objective lens of the confocal microscope. The laser power was fine-tuned, and the timing for opening and closing the laser shutter was regulated by the IR-LEGO system (IR-LEGO-100/mini/E, 808 nm of wavelength). The reduction in fluorescence caused by heating was measured and presented as *f*/*f*_0_. The increase in temperature was calculated by converting every 3.8% decrease in fluorescence into a 1 °C temperature increase, a correlation established in a previous work of Yamazaki et al.,^106^ with 37 °C considered as the initial temperature of the cells.

### Calcium Imaging on SH-SY5Y Cells

For the analysis of intracellular Ca^2+^ levels during NIR irradiation, cells were cultured in glass-bottom dishes and subjected to the previously described treatment regimen (24 h in a proliferation medium, followed by 72 h in a differentiation medium with or without 100 μg/mL of DiI-PDNPs). Thereafter, cells were stained with Fluo-4 AM (2 μM, Invitrogen) for 20 min at 37 °C. Then, samples were washed with DPBS and incubated in the imaging medium previously described for confocal microscopy time-lapse imaging, which was carried out with the same setting described in the previous paragraph. Cells were individually subjected to NIR laser irradiation (38.0 mW) for 50 ms or performing 20 repeated 50 ms irradiation with 100 ms intervals. Calcium channel involvement was confirmed by performing the experiment as previously described but with the addition of LaCl_3_ (Sigma-Aldrich), a well-known calcium channel blocker.^107^ Cells were preincubated with 10 μM LaCl_3_ for 30 min before stimulation.

Ca^2+^ source investigation was performed by incubating cultures during stimulation/imaging in phenol red-free Hanks’ Balanced Salt Solution without calcium and magnesium (HBSS, Fujifilm Chemicals) supplemented with 5 mM ethylene glycol tetraacetic acid (EGTA, BioWorld) or in phenol red-free Hanks’ Balanced Salt Solution with calcium and magnesium (HBSS+, Nacalai Tesque) supplemented with 1 μM thapsigargin (Fujifilm Chemicals). EGTA is a chelator that specifically targets calcium ions and is frequently utilized to investigate calcium role in different cellular functions,^108^ while thapsigargin is a guaianolide employed to deplete Ca^2+^ stores within the ER.^109^

### ACh Biosensor Transfection and NIR Stimulation in SH-SY5Y Cells

To monitor the release of ACh following stimulation, we utilized a genetically encoded acetylcholine sensor (GRAB_ACh3.0_).^46^ We obtained the plasmid encoding GRAB_ACh3.0_ gene from Addgene (plasmid # 121922); for mammalian cell expression, we amplified the whole DNA gene of GRAB_ACh3.0_ and inserted it into the pcDNA3.1(−) vector (Invitrogen) using *Bam*HI and *Hin*dIII restriction enzyme sites. We transformed the plasmid into *E. coli* DH5α competent cells (9057, Takara) and cultured them in Luria–Bertani (LB) broth medium supplemented with 100 μg/mL carbenicillin (Sigma) at 37 °C for 10–12 h. Subsequently, we performed plasmid purification. For transfection, SH-SY5Y cells were seeded at 10,000 cells/cm^2^ density in glass-bottom dishes and incubated for 24 h in proliferation medium. Cells were transfected with 1 μg of plasmid and 2.5 μg of PEI MAX (Polysciences) in Opti-MEM I (Gibco) for 6 h at 37 °C. Thereafter, cultures were washed with PBS and further incubated for 48 h with plain differentiation medium or differentiation medium supplemented with 100 μg/mL of DiI-PDNPs. Confocal microscopy imaging was conducted in the same setting previously described, while repeated NIR laser irradiation was performed as already outlined.

### Dopamine Level Rvaluation in SH-SY5Y Cells

To assess the impact of PDNPs on dopamine levels, neuron-like cells were cultured in a 24-well plate (Corning) at a density of 10,000 cells/cm^2^. After 24 h in a proliferation medium, cells were cultured for 72 h in differentiation medium with or without 100 μg/mL of PDNPs. Following this treatment, cell supernatants were collected and processed according to the manufacturer’s instructions for the selected assay kit (Dopamine ELISA Kit, ABCAM). The absorbance at 450 nm of the final reaction product was measured by using a Victor X3Multilabel Plate Reader (PerkinElmer).

### Heat-Induced Myotube Contraction

Confocal microscopy imaging was performed to evaluate the contraction produced by NIR irradiation of PDNP-treated myotubes. C2C12 cells were seeded at 10,000 cells/cm^2^ density onto a glass-bottom dish and incubated for 24 h in a proliferation medium. Thereafter, cells were incubated in plain differentiation medium or differentiation medium supplemented with 100 μg/mL of DiO-PDNPs. Cells were then incubated in DPBS supplemented with 5 μM CellTracker Orange (Invitrogen) and 5 μg/mL Hoechst, respectively, for cytoplasm and nuclei staining, and after 15 min at 37 °C washed three times with DPBS and incubated in the imaging medium previously described for time-lapse analysis. To inhibit myotube contraction, a myosin ATPase inhibitor, blebbistatin (Thermo Fisher, 25 μM), was added to the imaging medium. Confocal microscopy was conducted with the same setting as previously described. Myotubes were individually subjected to 10 s pulsed NIR laser irradiation (28.5 mW); time-lapse acquisitions were processed and compared by using the ImageJ software.

### Evaluation of Oxidative Stress Level Variation Following Thermal Stimulation

AuNSs, used as a positive control in these experiments, have been characterized for morphology, hydrodynamic diameter, ζ-potential, and NIR absorbance, following the same protocols previously described for PDNPs. To select the appropriate concentration of AuNSs to be compared with PDNPs, the photothermal conversion ability of the two nanostructures was assessed using a thermal imaging camera (A300, FLIR) during the irradiation of aqueous dispersions of PDNPs and AuNSs at concentrations ranging from 50 to 500 μg/mL.

Cells were cultured in μ-Plate 96-Well Black (Ibidi) at 10,000 cells/cm^2^ and subjected to the previously described procedure for cell differentiation; the following experimental conditions were analyzed: control, NIR, NIR + PDNPs (100.00 μg/mL), and NIR + AuNSs 50.00 μg/mL, were obtained from nanoComposix. Cells were subjected to laser irradiation for 5 min by using an NIR laser source able to irradiate multiple cells simultaneously. Following treatment, cells were incubated for 30 min with phenol red-free DMEM supplemented with 2.5 μM CellROX Green Reagent (Invitrogen) and subsequently detached. The relative fluorescence intensity of all of the experimental conditions was measured by flow cytometry (CytoFLEX platform, Beckman Coulter; λ_ex_ 488 nm, λ_em_ 525/40 nm). To further investigate variations in oxidative stress levels, cells were processed as previously described. In this case, however, cells were stained with CellROX before NIR laser stimulation and real-time monitored through confocal microscopy during laser stimulation. This approach allowed for the dynamic assessment of oxidative stress variations induced by PDNP-mediated photothermal stimulation.

### Proteomic Analyses

For both neuron-like cells and myotubes, cells were cultured in a 6-well plate (Corning) at 10,000 cells/cm^2^ and subjected to the previously described procedure for cell differentiation; the following experimental conditions were analyzed: control, NIR, PDNPs (100.00 μg/mL), and NIR + PDNPs (100.00 μg/mL). Cells were subjected to laser irradiation for 5 min using a NIR laser source able to irradiate multiple cells simultaneously.

Cells were then digested in RIPA Buffer (ThermoFisher), and for each replicate 50 μg of protein were processed. Cysteine residues were reduced/alkylated with a final concentration of 10 mM tris(2-carboxyethyl)phosphine (TCEP) and 30 mM chloroacetamide (95 °C for 15 min in the dark); after cooling at room temperature, samples were digested following the SP3 procedure.^110^ The concentration of the resulting peptides was checked using the Fluorometric Peptide Assay (ThermoFisher), and for each sample 300 ng of peptides were used. The proteomics analysis was conducted on a Thermo Exploris 480 orbitrap system coupled to a Dionex Ultimate 3000 nano-LC system. After trapping and desalting, the tryptic peptides were loaded on an Aurora C18 (75 × 250 mm, 1.6 μm particle size) nanocolumn (Ion Opticks, Fitzroy) and separated using a linear gradient of acetonitrile in water (both added with 0.1% formic acid), from 3% to 41% in 1 h, followed by column cleaning and reconditioning. The flow rate was set to 300 nL/min, the total run time was 1.5 h, and the injection volume was set to 1 μL. Peptides were analyzed in positive ESI mode, using a capillary voltage set to 2.0 kV. The RF lens was set to 40% and the AGC target was set to 300%. Data acquisition was performed in data independent mode (DIA) with a survey scan set from 400 to 1000 *m*/*z* at 120,000 resolution, followed by MS/MS acquisition of 60 *m*/*z* transmission windows, each having a fixed 10 Da width. MS/MS spectra were acquired in the HCD mode. All the collected MS/MS spectra were analyzed using Spectronaut (Version 18), by running a DirectDIA analysis against the reference *Homo sapiens* FASTA database (Tax ID: 9606 reporting 51548 reviewed entries). Cysteine carbamidomethylation was selected as a fixed modification; acetylation of protein N-term and methionine oxidation were selected as variable modifications. Positive protein identifications were retained at a 1% false discovery rate (FDR) threshold, and at least two peptides were used for protein quantification.

### Calcium Imaging on *Drosophila melanogaster* Brain

Flies (*D. melanogaster*) were bred by feeding with standard fly medium following normal 12 h light/12 h dark conditions at 25 °C. A jGCaMP7c (BL#79030) calcium indicator was expressed in neurons expressing the *fruitless* gene (*fru*, BL#66696) through the GAL4/UAS system. Male flies were collected and maintained in a group of 10–20 flies per vials and used 6–7 days after eclosion. Brains were dissected in Ca^2+^-free adult hemolymph-like saline (AHLS), and the blood–brain barrier (BBB) was decomposed with papain (10 U/mL, Worthington Biochemical Corporation) for 15 min at RT. Before the observation, control brains remained untreated, while other brains were treated for 30 min with 100 μg/mL DiI-PDNPs, 100 μg/mL DiI-PDNPs, and 100 nM donepezil hydrochloride (TCI Chemicals), or with 100 μg/mL DiI-PDNPs and 5 μM α-BTX (Alomone Laboratories). During observation, the fly brains were soaked in AHLS supplemented with 108 mM NaCl, 5 mM KCl, 2 mM CaCl_2_, 8.2 mM MgCl_2_, 4 mM NaHCO_3_, 1 mM NaH_2_PO_4_, 5 mM trehalose, 10 mM sucrose, and 5 mM HEPES. Confocal microscopy imaging was conducted with the same setting described in the previous paragraph while undergoing NIR laser irradiation (38 mW) for 10 s.

### Statistical Analysis

Statistical analysis was conducted using *R* software, and each experimental condition was compared with its respective control. Normal distribution was assessed using the Shapiro–Wilk normality test. For normally distributed data, analysis was performed using ANOVA followed by the LSD posthoc test with Bonferroni’s correction, and the results were presented as average ± standard deviation. Non-normally distributed data were analyzed using the Kruskal–Wallis test followed by the Wilcox posthoc test with the Holm correction, and the results were expressed as median ± 95% confidence interval. Each experiment was conducted in triplicate (*n* = 3), unless otherwise specified.

For proteomics, please refer to the relevant experimental section.

## Data Availability

Proteomics data are available via ProteomeXchange with identifier PXD061533. All other data are available from the authors upon reasonable request.

## Supporting Information Available

PDNP stability evaluation; PDNP degradation evaluation; nanoparticle/cell interaction analysis for SH-SY5Y and C2C12 cells; colocalization with organelles in SH-SY5Y and C2C12 cells; neuronal differentiation analysis; myotube differentiation analysis; calcium imaging upon 50 ms of laser irradiation; calcium transient production on adjacent cells; dopamine levels quantification; AuNS characterization; proteomic analyses; ex vivo *D. Melanogaster* experiment summary (PDF)
Time-lapse video of a subcellular contraction in C2C12 myotubes (AVI)

## Supplementary Material

nn5c04181_si_001.pdf

nn5c04181_si_002.avi
