# Supplementary material for: Cellular Activity Modulation Mediated by Near Infrared-Irradiated Polydopamine Nanoparticles: In Vitro and Ex Vivo Investigation
Source: ACS Nano. 2025 Apr 24;19(17):16267–86. doi: 10.1021/acsnano.5c04181 (PMC12060647; doi:10.1021/acsnano.5c04181)
Supplement: Supplementary file 1 — nn5c04181_si_001.pdf [file nn5c04181_si_001.pdf]

SUPPORTING INFORMATION FOR

Cellular activity modulation mediated by NIR-irradiated polydopamine nanoparticles: *In vitro* and *ex vivo* investigation

Alessio Carmignani[a,], Takeru Yamazaki[b,*], Matteo Battaglini[a], Cong Quang Vu[b], Attilio Marino[a], Seika Takayanagi-Kiya[c], Taketoshi Kiya[c], Andrea Armirotti[d], Andrea Di Fonzo[d], Satoshi Arai[b,*,#], Gianni Ciofani[a,*,#]*

a. Istituto Italiano di Tecnologia, Smart Bio-Interfaces, Viale Rinaldo Piaggio 34, 56025 Pontedera, Italy

b. Kanazawa University, WPI Nano Life Science Institute, Kakuma-machi, 920-1192 Kanazawa, Japan

c. Kanazawa University, Graduate School of Natural Science & Technology, Kakuma-machi, 920-1192 Kanazawa, Japan

d. Istituto Italiano di Tecnologia, Analytical Chemistry Facility, Via Morego 30, 16163 Genova, Italy

*Corresponding Authors: alessio.carmignani@iit.it; takeru-yamazaki@stu.kanazawa-u.ac.jp; satoshi.arai@staff.kanazawa-u.ac.jp; gianni.ciofani@iit.it

#Equally Contributing Authors

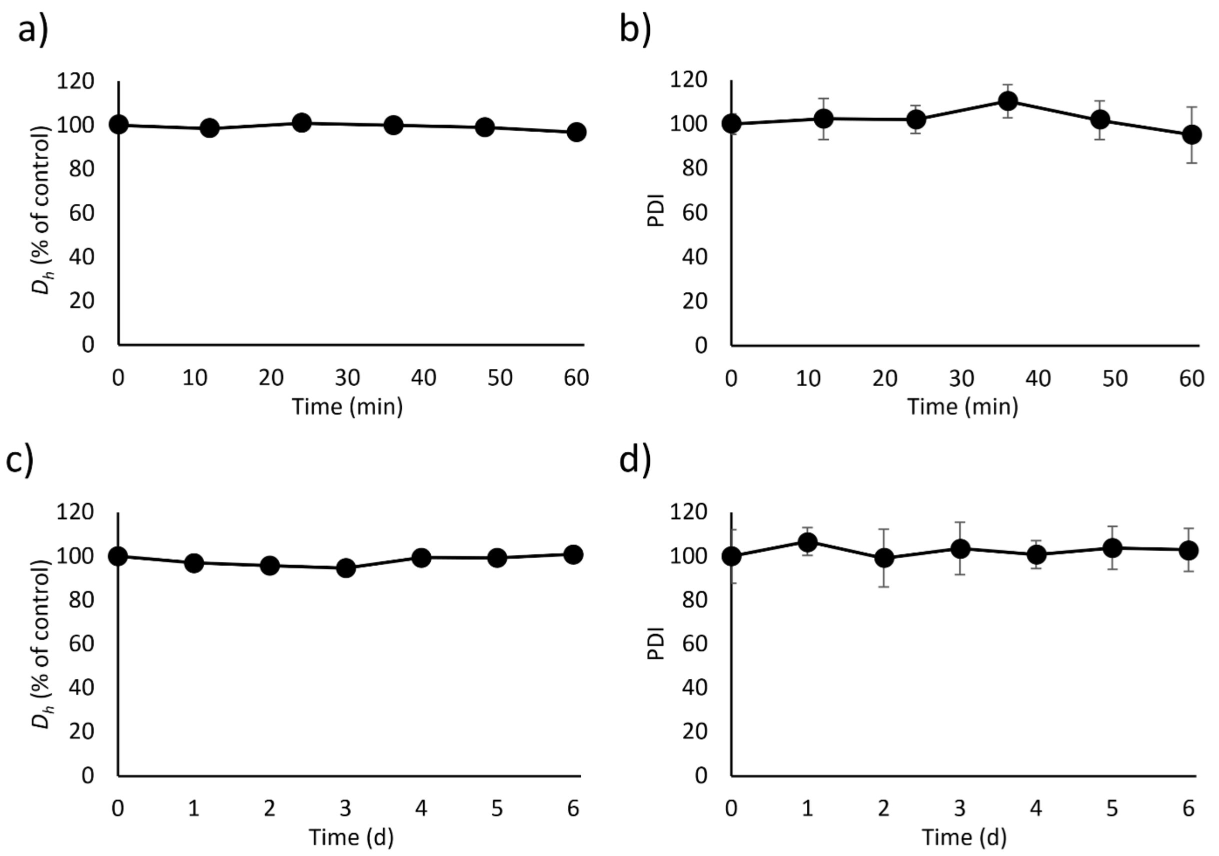

Figure S1. PDNP stability assay performed in cell culture medium. Analysis of a, c) D_h and b, d) PDI values over a, b) 1 h and c, d) 6 days (n = 3).

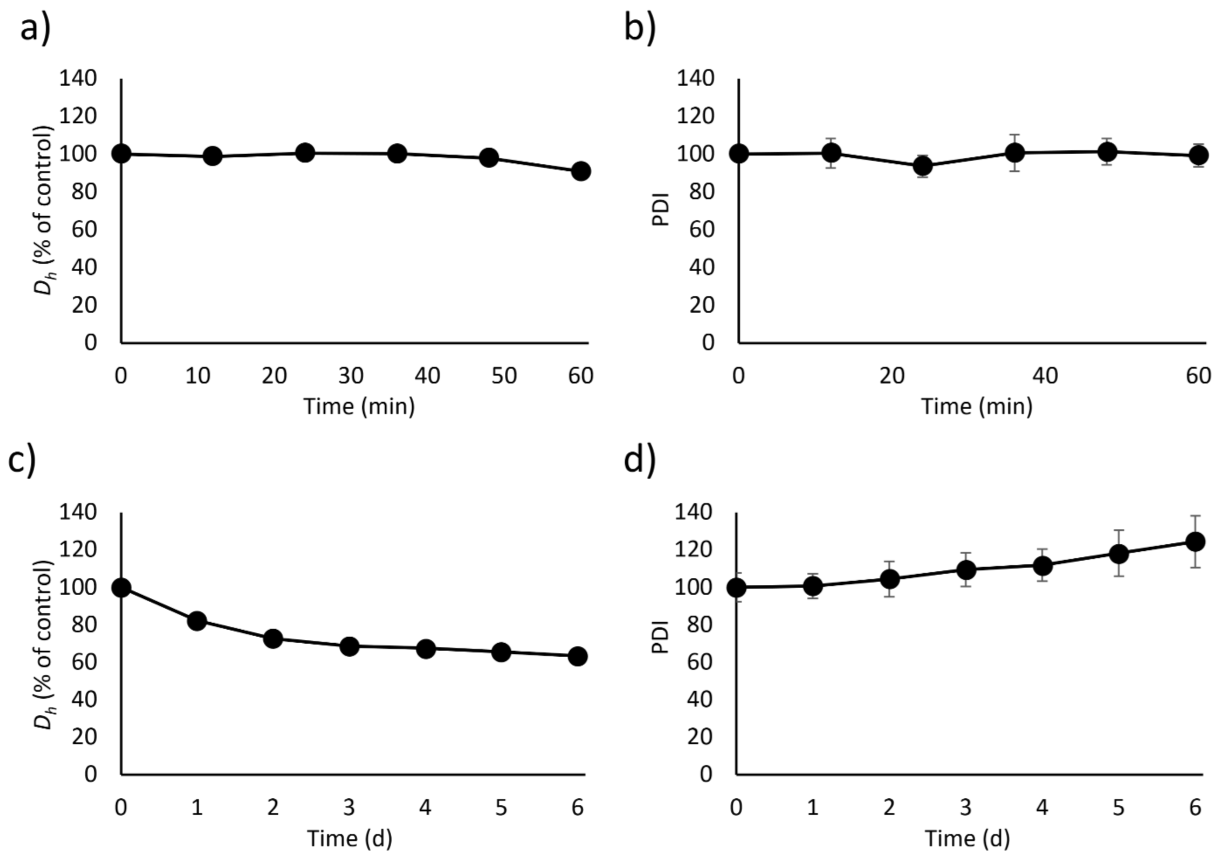

Figure S2. PDNP degradation performed analysis following incubation with cathepsin B. Analysis of a, c) D_h and b, d) PDI values over a, b) 1 h and c, d) 6 days (*n* = 3).

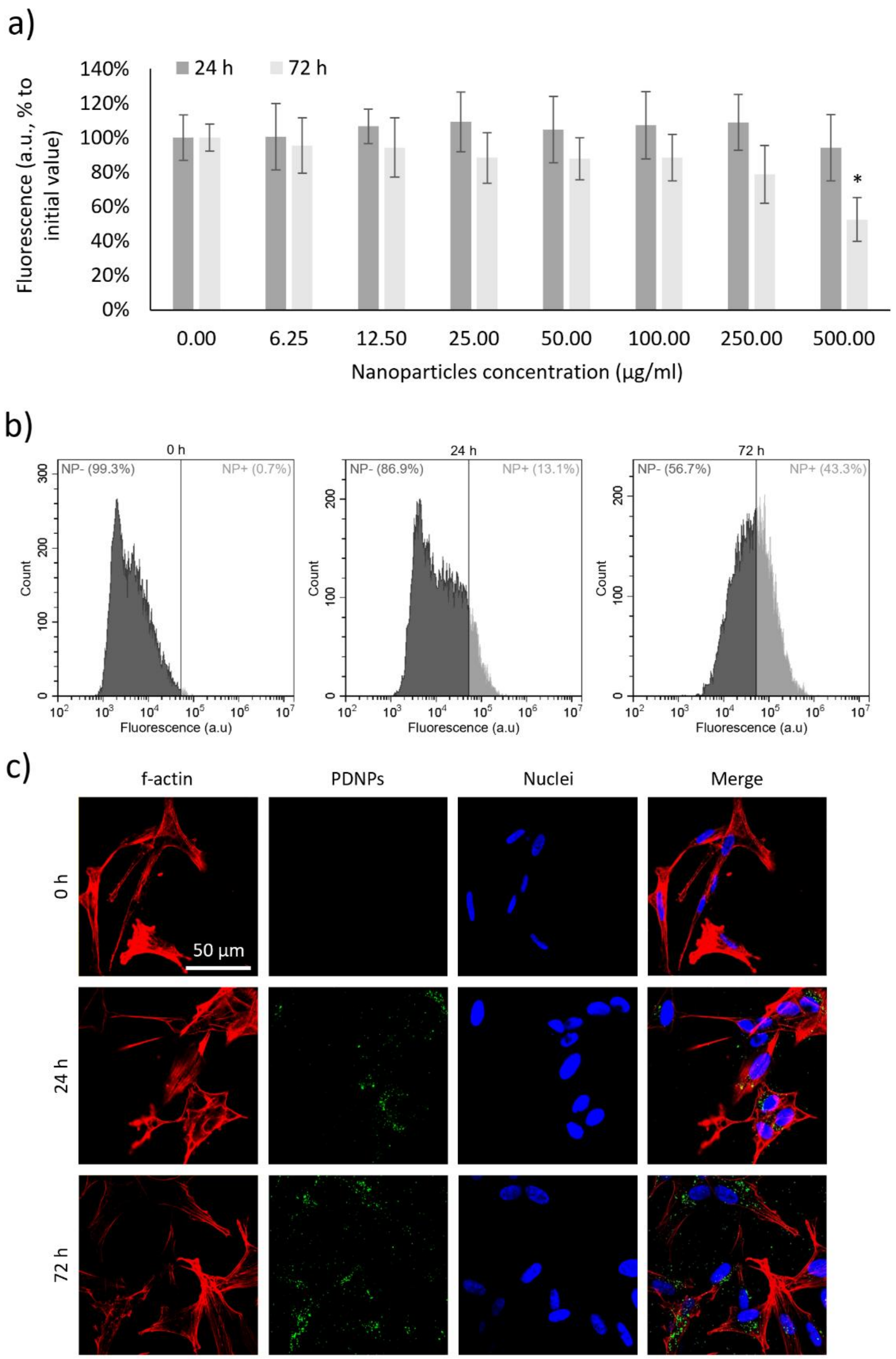

Figure S3. Nanoparticle/cell interaction assessments. a) PicoGreen assay performed on differentiated SH-SY5Y cells after NIR laser stimulation (n = 6, * $p < 0.05$); b) representative flow cytometry data and c) representative confocal microscopy acquisitions of PDNP uptake by differentiated SH-SY5Y cells (f-actin in red, DiO-PDNPs in green, nuclei in blue).

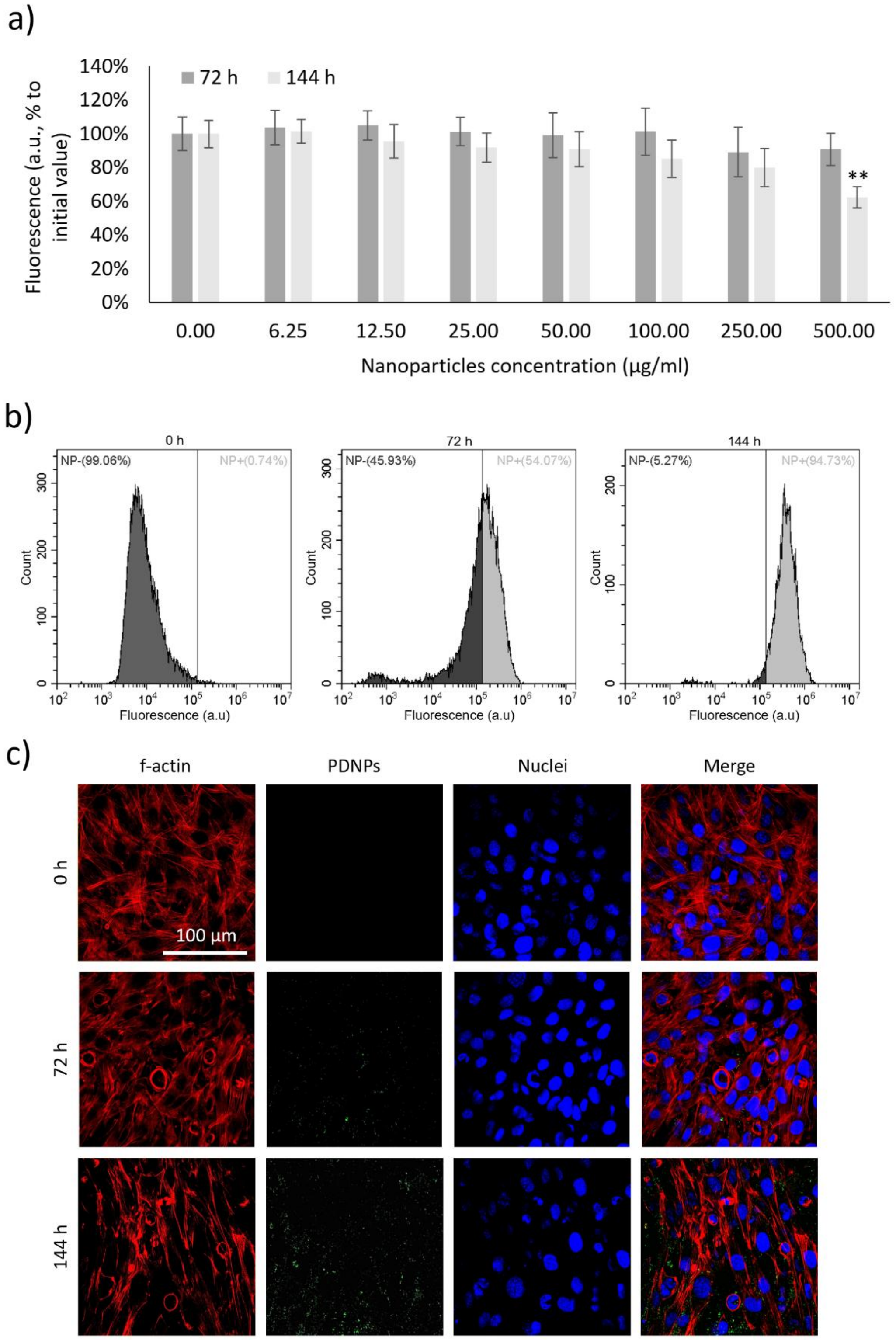

Figure S4. Nanoparticle/cell interaction assessments. a) PicoGreen assay performed on differentiated C2C12 cells after NIR laser stimulation (n = 6, * p < 0.05); b) representative flow cytometry data and c) representative confocal microscopy acquisitions of PDNP uptake by differentiated C2C12 cells (f-actin in red, DiO-PDNPs in green, nuclei in blue).

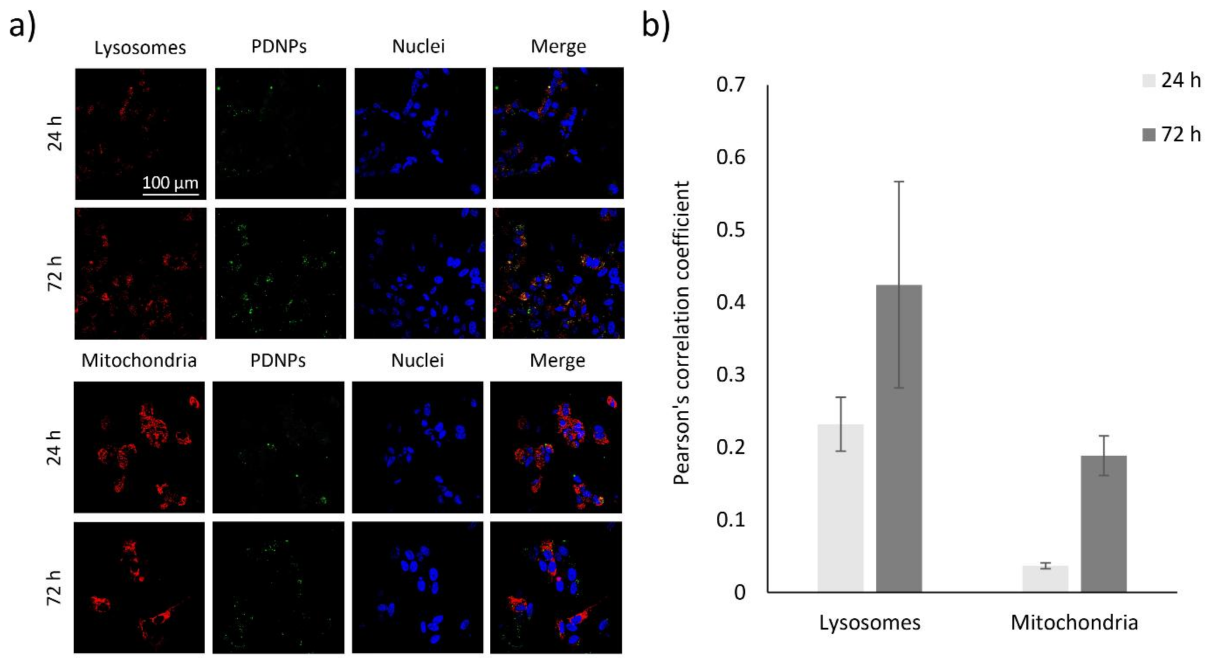

Figure S5. Analysis of the intracellular localization of DiO-PDNPs in differentiated SH-SY5Y cells. a) Representative confocal images showing the intracellular localization of DiO-PDNPs with respect to mitochondria and lysosomes (organelles in red, DiO-PDNPs in green, nuclei in blue). b) Pearson's correlation coefficients analysis between DiO-PDNP and organelle fluorescence signals.

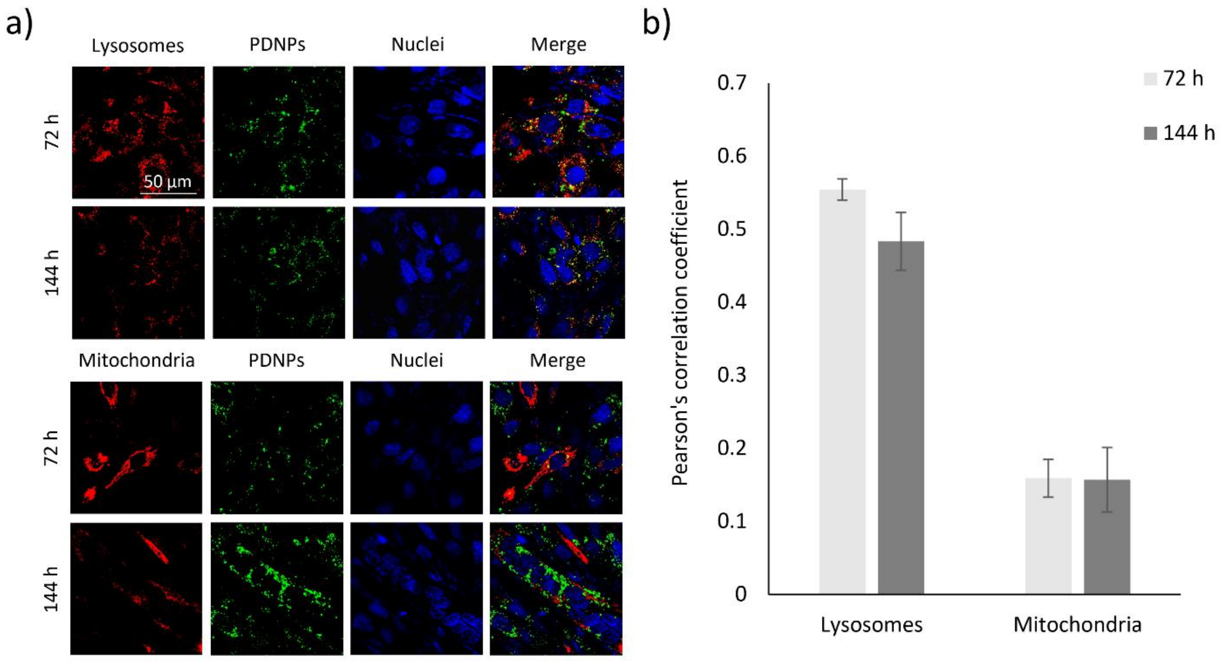

Figure S6. Analysis of the intracellular localization of DiO-PDNPs in differentiated C2C12 cells. a) Representative confocal images showing the intracellular localization of DiO-PDNPs with respect to mitochondria and lysosomes (organelles in red, DiO-PDNPs in green, nuclei in blue). b) Pearson's correlation coefficients analysis between DiO-PDNP and organelle fluorescence signals.

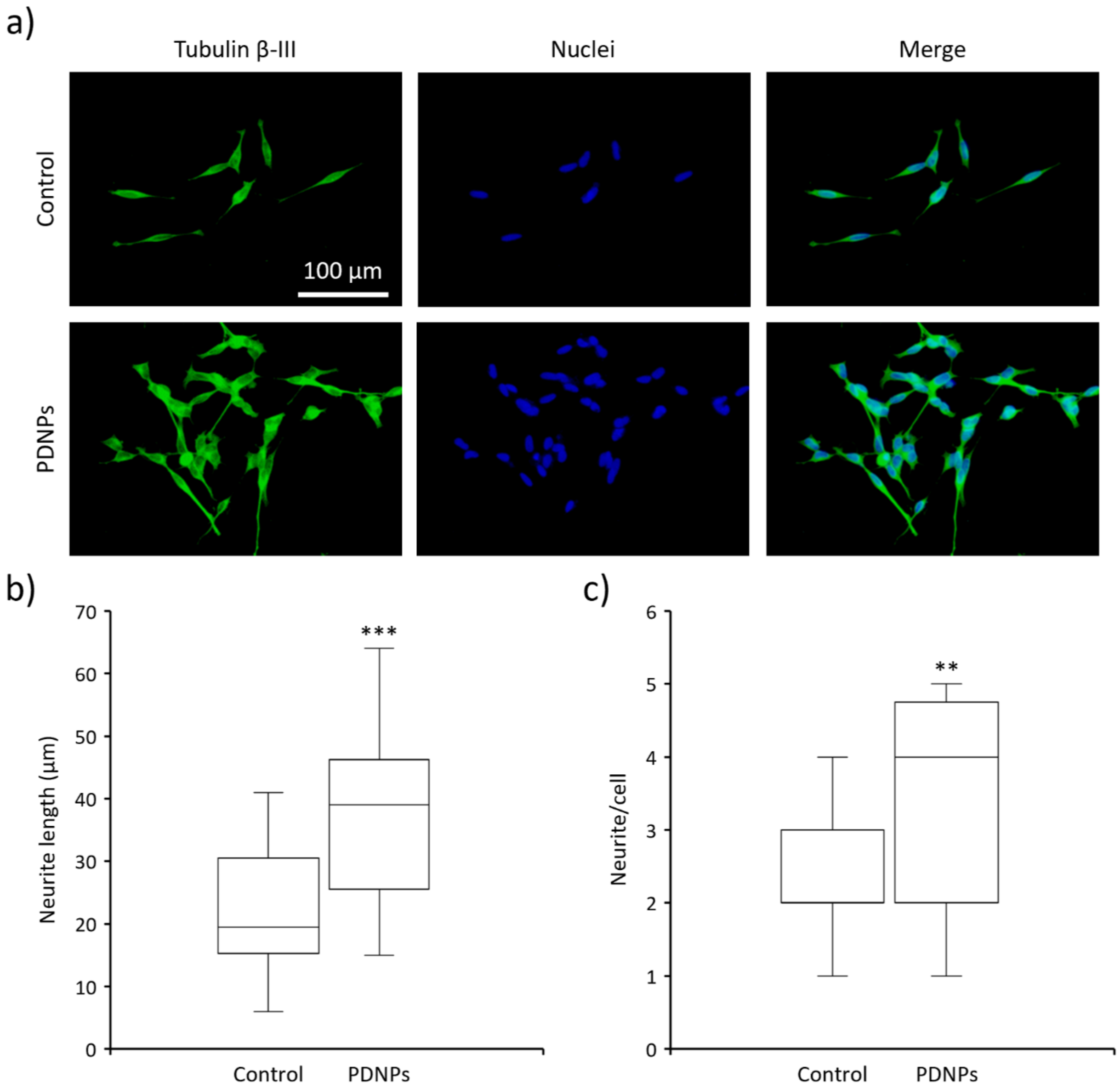

Figure S7. Analysis of the PDNP effect on neurite outgrowth of differentiating SH-SY5Y cells. a) Representative epifluorescence images of SH-SY5Y treated or not with PDNPs (tubulin β-III in green, nuclei in blue). b) Comparison of the median neurite length with or without PDNP treatment (n = 30, *** p < 0.001). c) Comparison of the neurite/cell ratio with or without PDNP treatment (n = 30, ** p < 0.01).

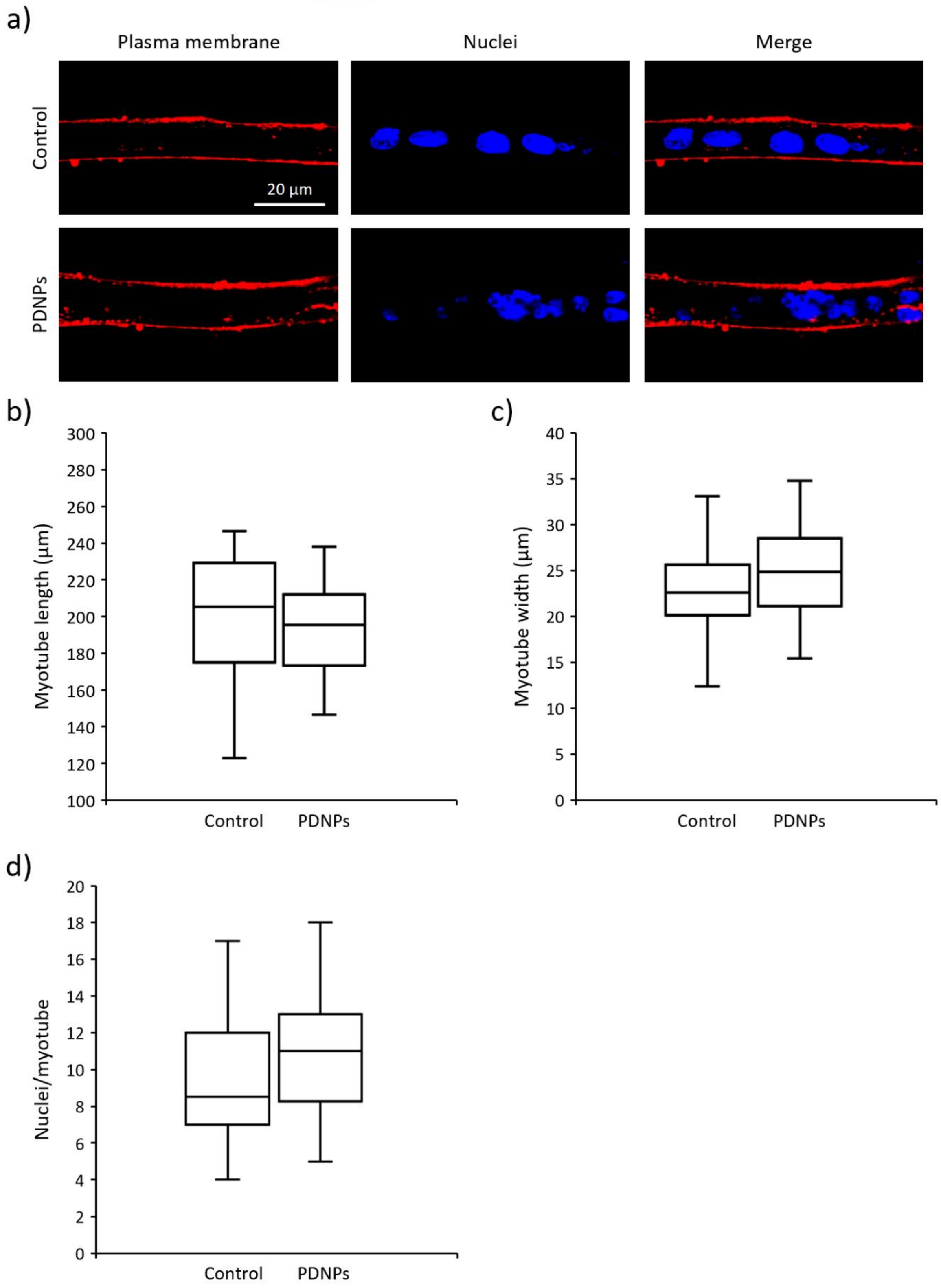

Figure S8. Analysis of the PDNP effect on myotube differentiation. a) Representative confocal microscopy images of C2C12 treated or not with PDNPs (plasma membrane in red, nuclei in blue). Comparison of the myotube b) length and c) width in absence or presence of PDNPs (*n* = 30). d) Comparison of the nuclei/myotube ratio in absence or presence of PDNPs (*n* = 30).

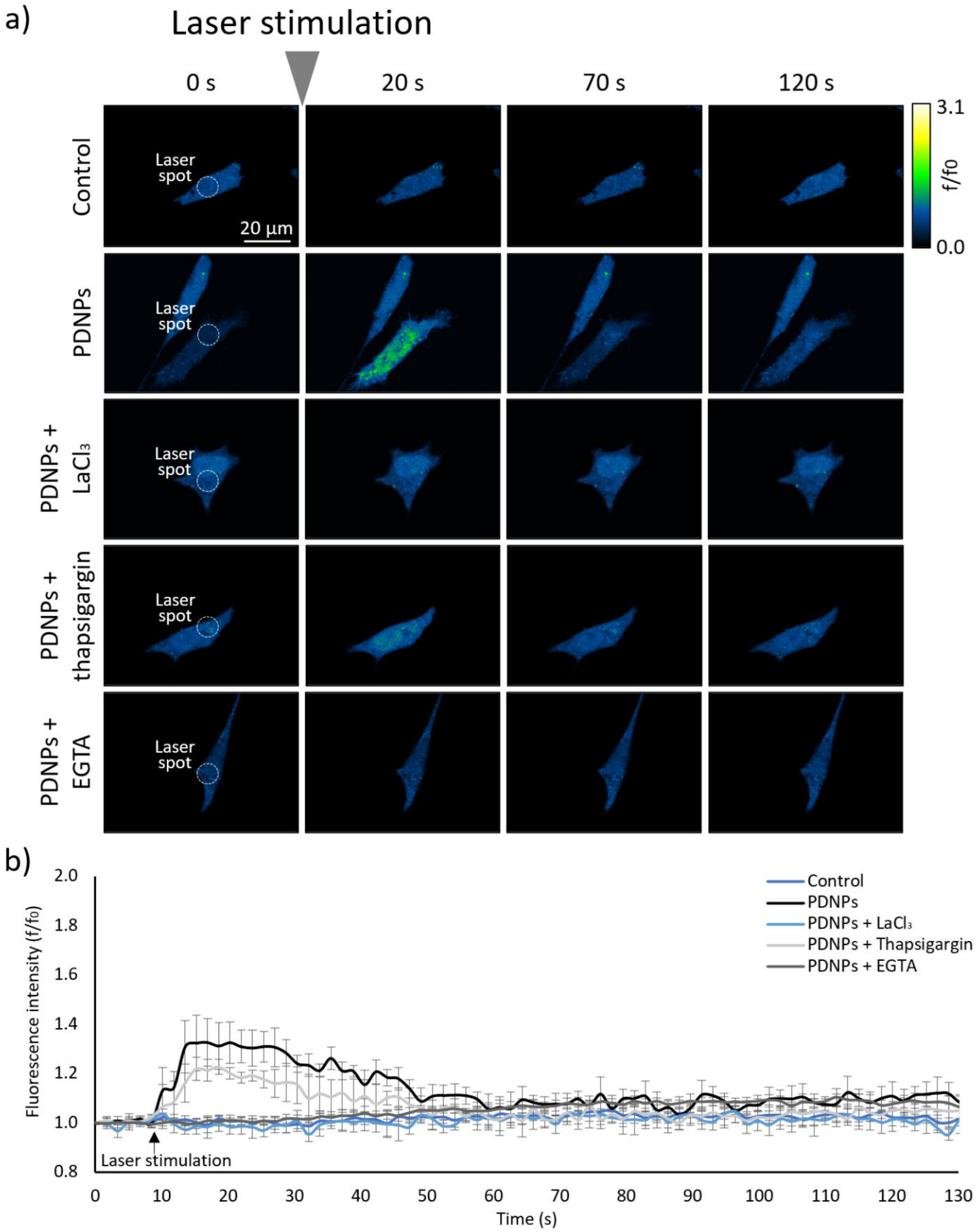

Figure S9. Calcium imaging on SH-SY5Y cells subjected to a single 50 ms NIR laser stimulation. a) Representative time frame images. b) Time course of the variation of cell fluorescence levels is indicative of calcium concentration (n = 10).

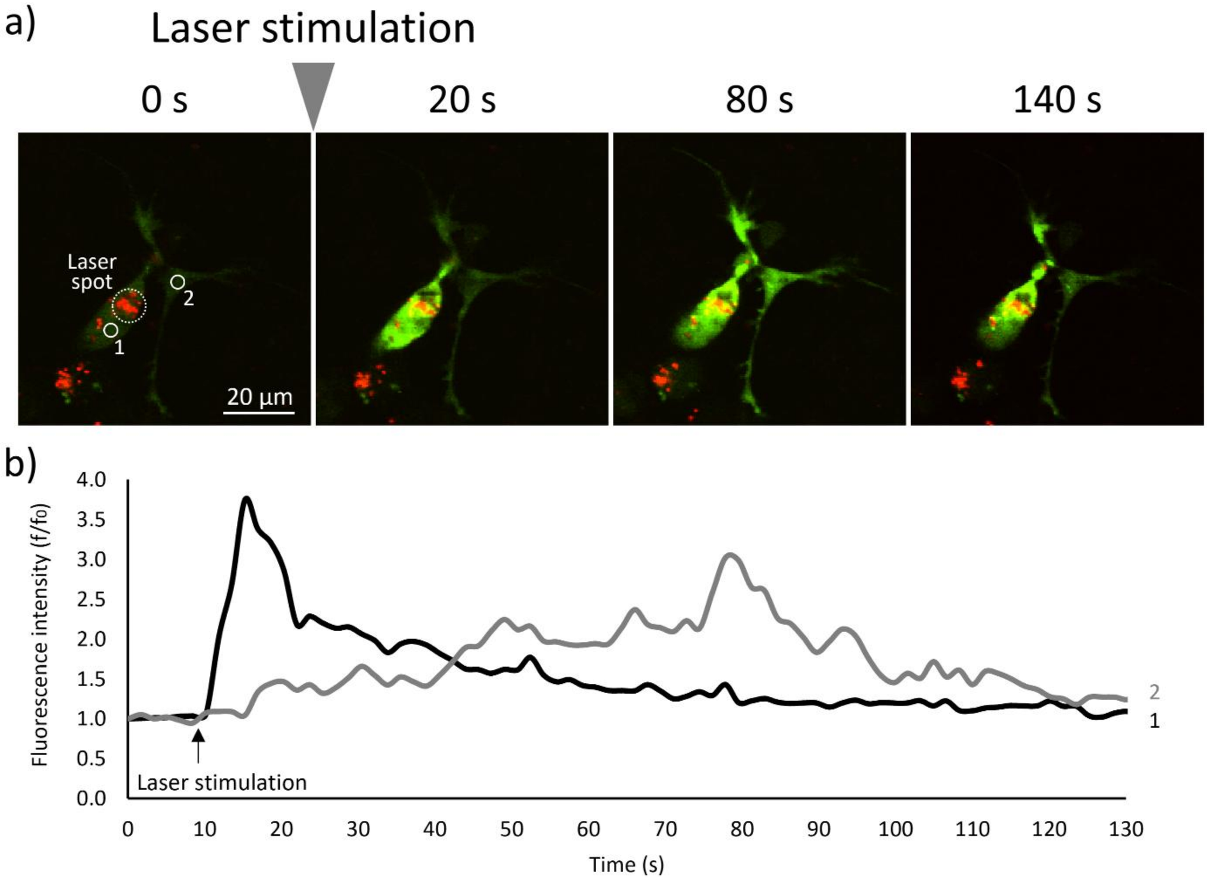

Figure S10. Calcium imaging analyses performed on adjacent cells. a) Representative time frames of calcium imaging performed on differentiated SH-SY5Y cells during NIR laser stimulation (DiI-PDNPs in red, calcium in green). b) Time course of the variation of cell fluorescence levels, indicative of calcium concentration, during NIR laser stimulation. ROI 1 shows the calcium transient generated in the irradiated cell, while ROI 2 shows the subsequent calcium transient produced in the adjacent cell.

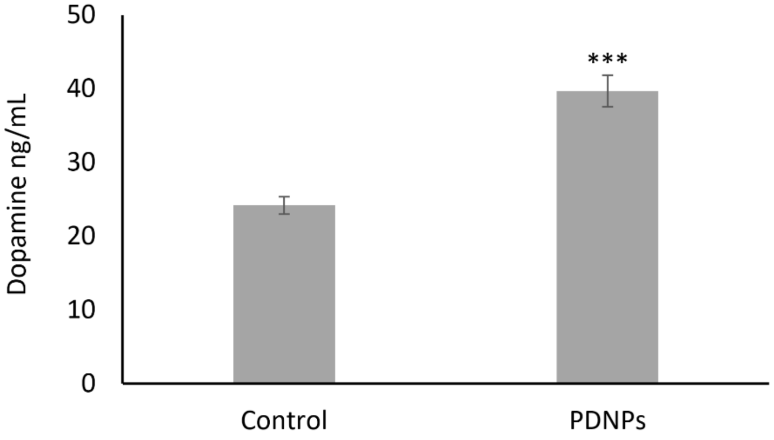

Figure S11. Dopamine levels quantification in differentiated SH-SY5Y cultures treated with PDNPs (n = 3, *** $p < 0.001$).

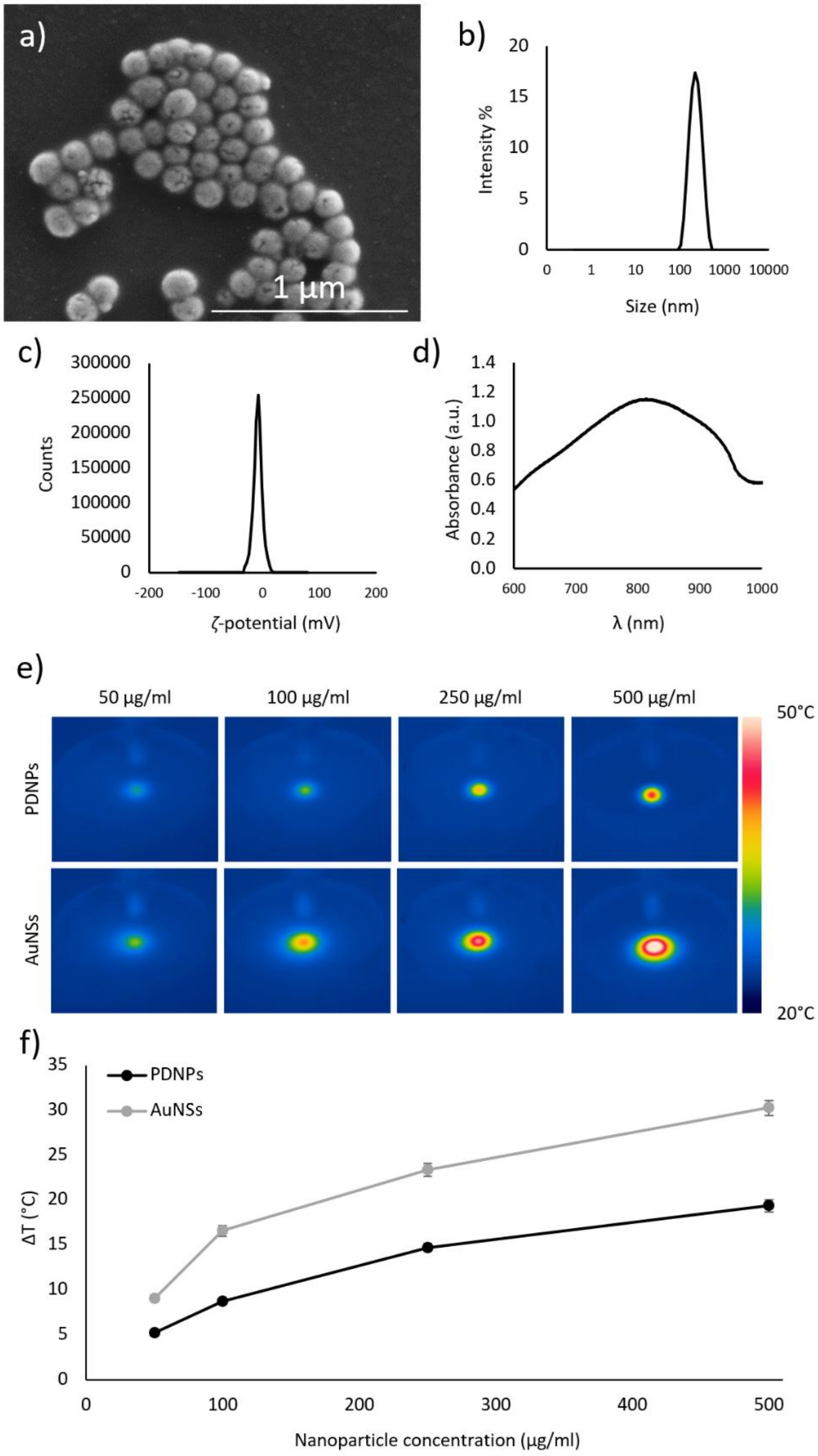

Figure S12. AuNS characterization. a) Representative SEM image; b) hydrodynamic diameter distribution; c) ζ-potential analysis; d) NIR absorption spectrum; e) representative thermal images captured after 5 min of NIR irradiation of aqueous dispersions containing different concentrations of PDNPs and AuNSs; f) temperature increment recorded along 5 min of NIR irradiation.

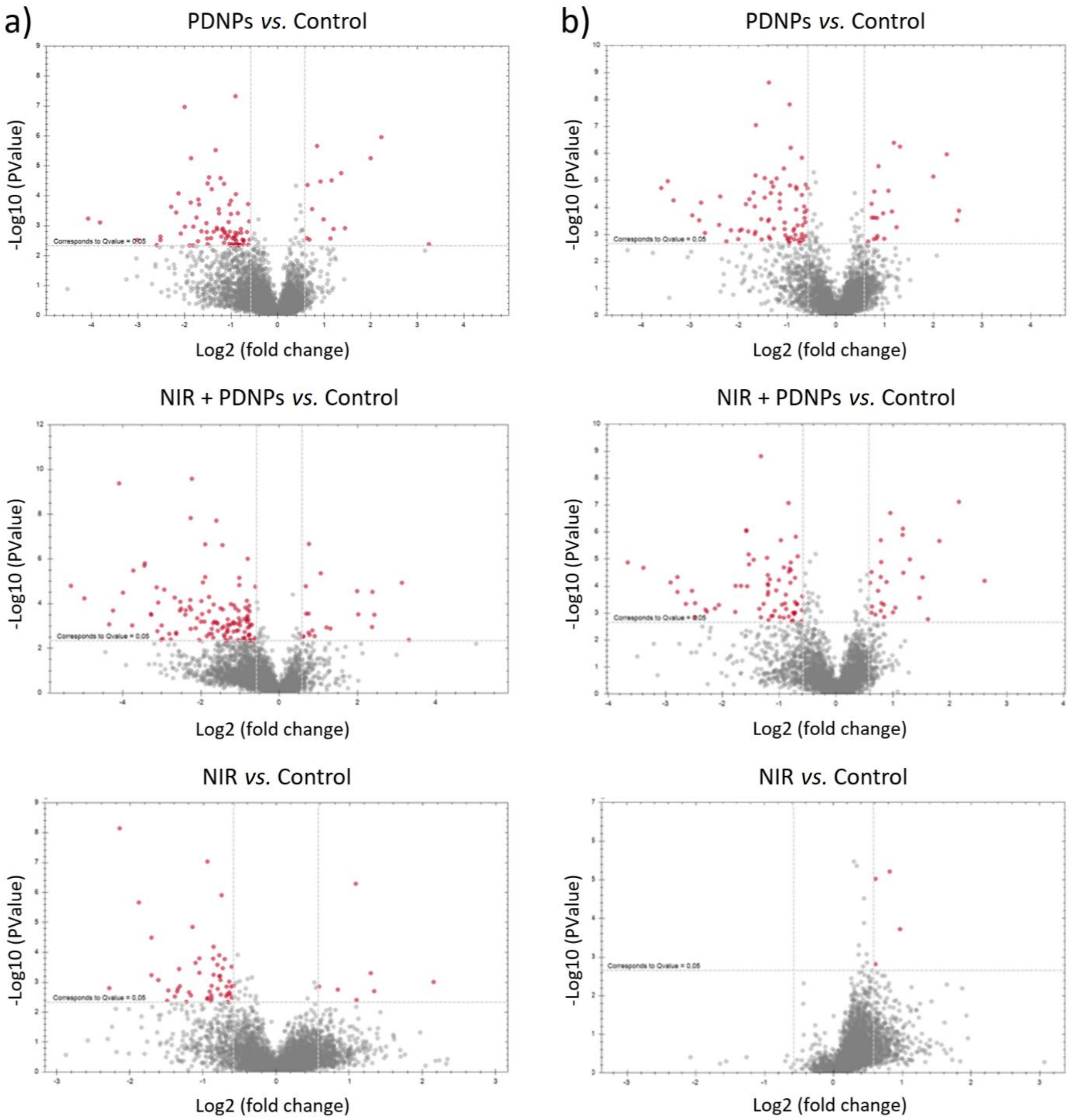

Figure 13. Volcano plots of differentially expressed proteins. Plots depicting differentially expressed proteins in a) neurons and b) myotubes. The *x*-axis represents the log2 fold change, while the *y*-axis shows the -log10 *p*-value. Red dots indicate statistically significant upregulated and downregulated proteins.

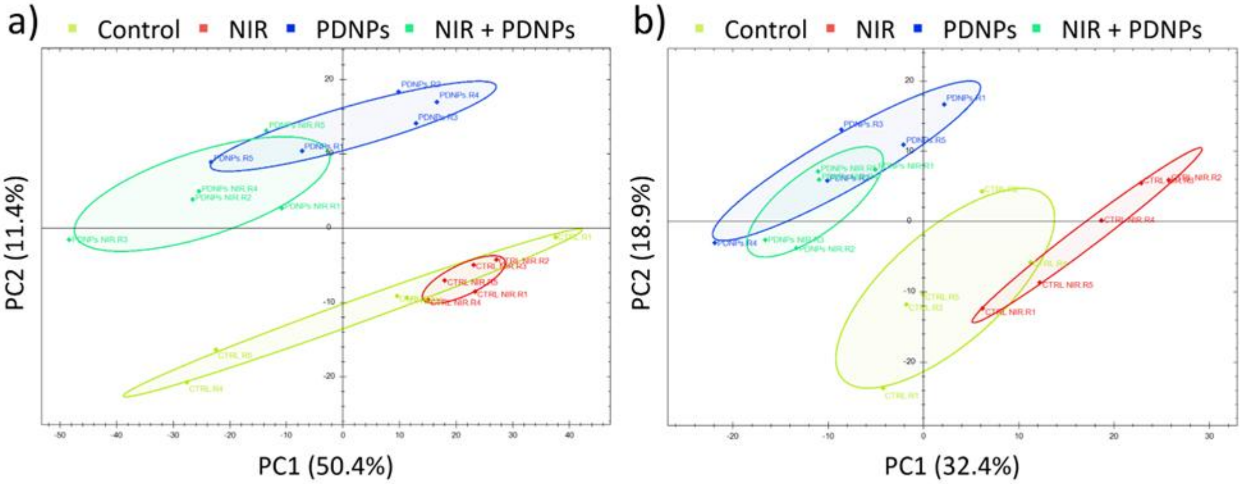

Figure 14. Principal component analysis (PCA) of proteomic data. Plots illustrating the variance in proteomic profiles among experimental conditions in a) neurons and b) myotubes.

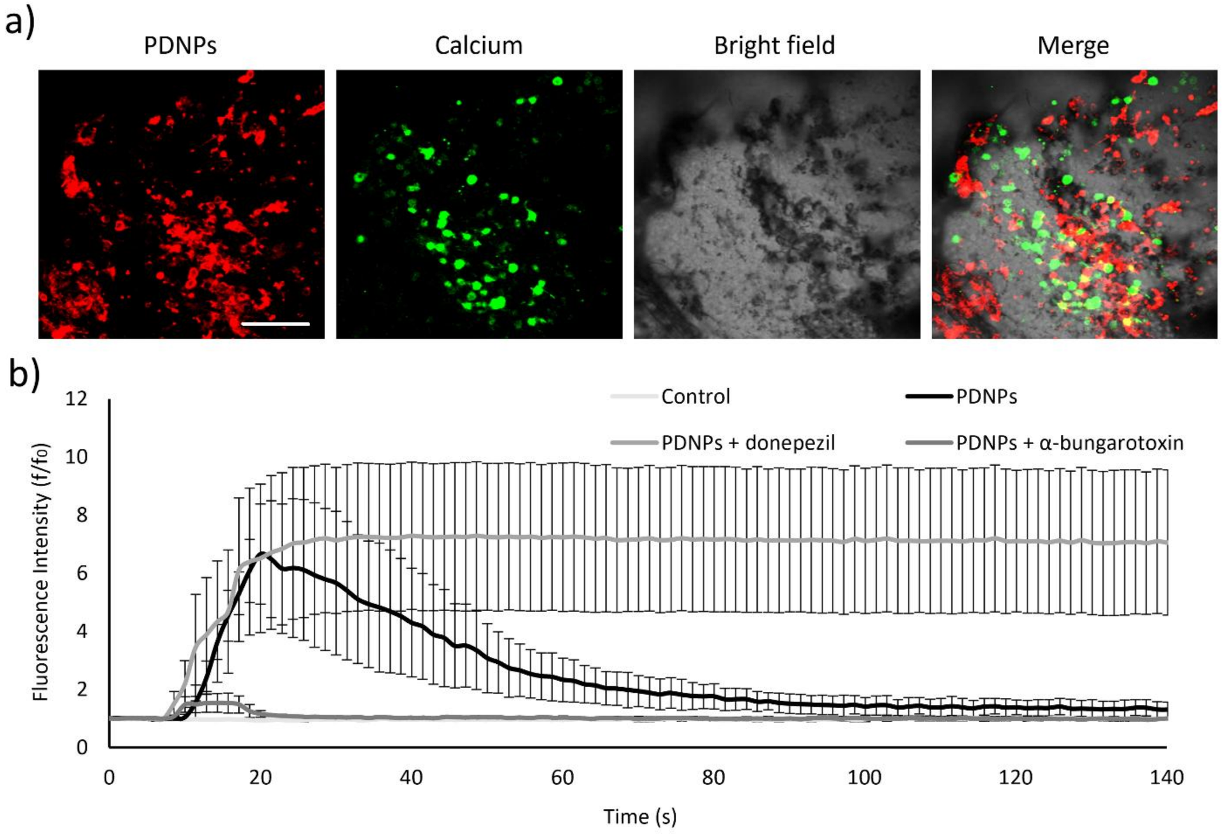

Figure S15. Summary the calcium imaging measurements performed on *Drosophila* brains. a) Representative confocal image of a *Drosophila* brain treated with PDNPs (DiI-PDNPs in red, jGCaMP7c in green). b) Time course of fluorescence levels, indicative of calcium concentration.

Video S1. Representative time-lapse video of a subcellular contraction in C2C12 myotubes in response to the NIR + PDNP stimulation. Cytoplasm in red, DiO-PDNPs in green.
